# Transcriptomic and Proteomic Insights into Host Immune Responses in Pediatric Severe Malarial Anemia: Dysregulation in HSP60-70-TLR2/4 Signaling and Altered Glutamine Metabolism

**DOI:** 10.3390/pathogens13100867

**Published:** 2024-10-03

**Authors:** Clinton O. Onyango, Samuel B. Anyona, Ivy Hurwitz, Evans Raballah, Sharely A. Wasena, Shamim W. Osata, Philip Seidenberg, Benjamin H. McMahon, Christophe G. Lambert, Kristan A. Schneider, Collins Ouma, Qiuying Cheng, Douglas J. Perkins

**Affiliations:** 1 Department of Biomedical Sciences and Technology, School of Public Health and Community Development, Maseno University, Maseno 40100, Kenya; clintononyango4@yahoo.com (C.O.O.); sharleyawasena@gmail.com (S.A.W.); shamimosata@gmail.com (S.W.O.); collinouma@yahoo.com (C.O.); 2 Center for Global Health, Internal Medicine, University of New Mexico, Albuquerque, NM 87131, USA; ihurwitz@salud.unm.edu (I.H.); pseidenberg@salud.unm.edu (P.S.); mcmahon@lanl.gov (B.H.M.); kaschneider@salud.unm.edu (K.A.S.); 3 Kenya Global Health Programs, University of New Mexico, Kisumu and Siaya 40100, Kenya; sbonuke@gmail.com (S.B.A.); eraballah@hotmail.com (E.R.); 4 Department of Medical Biochemistry, School of Medicine, Maseno University, Maseno 40100, Kenya; 5 Department of Medical Laboratory Sciences, School of Public Health Biomedical Sciences and Technology, Masinde Muliro University of Science and Technology, Kakamega 50100, Kenya; 6 Department of Emergency Medicine, School of Medicine, University of New Mexico, Albuquerque, NM 87131, USA; 7 Theoretical Biology and Biophysics Group, Los Alamos National Laboratory, Theoretical Division, Los Alamos, NM 87545, USA; 8 Department of Internal Medicine, Division of Translational Informatics, University of New Mexico, Albuquerque, NM 87131, USA

**Keywords:** RNA-seq, proteomics, host immune response, childhood innate immunity, differential gene expression, heat shock proteins, toll-like receptors, glutamine transporters, glutamine synthetase

## Abstract

Severe malarial anemia (SMA, Hb < 6.0 g/dL) is a leading cause of childhood morbidity and mortality in holoendemic *Plasmodium falciparum* transmission zones. This study explored the entire expressed human transcriptome in whole blood from 66 Kenyan children with non-SMA (Hb ≥ 6.0 g/dL, n = 41) and SMA (n = 25), focusing on host immune response networks. RNA-seq analysis revealed 6862 differentially expressed genes, with equally distributed up-and down-regulated genes, indicating a complex host immune response. Deconvolution analyses uncovered leukocytic immune profiles indicative of a diminished antigenic response, reduced immune priming, and polarization toward cellular repair in SMA. Weighted gene co-expression network analysis revealed that immune-regulated processes are central molecular distinctions between non-SMA and SMA. A top dysregulated immune response signaling network in SMA was the HSP60-HSP70-TLR2/4 signaling pathway, indicating altered pathogen recognition, innate immune activation, stress responses, and antigen recognition. Validation with high-throughput gene expression from a separate cohort of Kenyan children (n = 50) with varying severities of malarial anemia (n = 38 non-SMA and n = 12 SMA) confirmed the RNA-seq findings. Proteomic analyses in 35 children with matched transcript and protein abundance (n = 19 non-SMA and n = 16 SMA) confirmed dysregulation in the HSP60-HSP70-TLR2/4 signaling pathway. Additionally, glutamine transporter and glutamine synthetase genes were differentially expressed, indicating altered glutamine metabolism in SMA. This comprehensive analysis underscores complex immune dysregulation and novel pathogenic features in SMA.

## 1. Introduction

Malaria remains a significant threat to public health globally, with an estimated 249 M cases and 608,000 deaths, with the majority (80%) of malaria-related mortality occurring in children under five years in the African Region [1]. In holoendemic *Plasmodium falciparum* transmission regions, such as western Kenya, children are vulnerable to severe malaria anemia [SMA, hemoglobin (Hb) < 6.0 g/dL], a primary manifestation of severe malaria. In contrast, cerebral malaria (CM) is rare in the region [2,3]. The etiology of SMA includes hemolysis [destruction of infected and uninfected red blood cells (RBCs)], splenic sequestration of RBCs, dyserythropoiesis, and bone marrow suppression, often complicated by co-infection with other pathogens [4]. Our previous studies showed that genetic variation and dysregulation in innate immune response genes, such as *C3*, *C5*, *CSF2*, *IFN-γ*, *IL-1β*, *IL-7*, *IL-10*, *IL-12*, *LAIR1*, *NCR3*, and *RANTES*, play a crucial role in the pathogenesis of SMA [2,5,6,7,8,9,10,11].

Studies from our group have also shown that additional innate immune response genes were altered in children with SMA, including down-regulation of heat shock protein 70 (HSP70) transcripts driven by leukocytic phagocytosis of malarial pigment [hemozoin (*Pf*Hz)] [12]. Human HSPs are a large superfamily of molecular chaperones that are cytoprotective and anti-inflammatory through their ability to correct and avoid misfolded proteins for proper proteostasis within cellular compartments [13]. Family members include HSP60 (encoded by *HSPD1*) and HSP70, with distinct members encoded by separate genes [HSP70-1 (*HSPA1A*), HSP70-2 (*HSPA1B*), HSP70-4 (*HSPA4*), HSP70-4L (*HSPA4L*), and HSP70-5 (*HSPA5*)], among others [14]. HSPs are vital for maintaining cellular homeostasis under physiological and stress conditions such as hypoxia and heat shock [15]. HSPs are known immunomodulants that regulate the production and release of various cytokines (e.g., IL-1β, IL-6, IL-10, IL-12, TNF-α, and IFN-γ) [16,17], a group of inflammatory mediators we have shown that are dysregulated in children with SMA [4,5,18,19,20,21,22,23,24]. Moreover, HSPs act as danger-associated molecular patterns (DAMPs), activating signaling cascades when released extracellularly into circulation by necrotic and stressed cells [25,26]. Extracellular HSP60 and HSP70 can activate immune responses by binding to toll-like receptors (TLR) 2 and 4, essential pattern recognition receptors (PRRs) of innate immunity responsible for recognizing pathogen-associated molecular patterns (PAMPs) and DAMPs [27,28,29]. Known PAMPs in malaria include *Plasmodium* glycosylphosphatidylinositols (GPIs) that bind avidly to TLR2 and less stringently to TLR4 [30,31]. Upon recognizing PAMPs, TLRs form homodimers or heterodimers to transduce the TLR 2/4 signaling through the myeloid differentiation primary response 88 (MyD88) pathway for activation of nuclear factor kappa-light-chain-enhancer of activated B cells (NF-κB) and subsequent production of pro-inflammatory cytokines and type I interferons [32,33]. TLR4 can also signal through the TIR-domain-containing adapter-inducing interferon-β (TRIF) pathway to activate interferon regulatory factors (IRFs) and produce type I interferons [32,33]. When TLR2/4 is activated, extracellular HSP60 and HSP70 can enhance antigen presentation by increasing the expression of major histocompatibility complex (MHC) II molecules, thereby coordinating innate and adaptive immune responses [25,34,35,36,37,38].

L-glutamine (GLN), the most abundant amino acid in the human body, is a key molecule for up-regulating HSP70 [39]. GLN is a conditionally essential amino acid required for proper immune cell function, regulation of cytokine balance, and antioxidant defense [40]. We have previously shown that circulating GLN is significantly reduced in children with SMA and that low GLN levels strongly predict SMA development [12]. These investigations further demonstrated that GLN treatment of peripheral blood mononuclear cells (PBMCs) overcame *Pf*Hz-induced suppression of HSP70 gene transcription and translation, reduced NF-κB activation, and mitigated the overexpression of IL-1β, IL-6, and TNF-α [12]. Moreover, recent investigations demonstrated that supplementation of cultured RBCs with amino acids, including GLN, reduces the oxidative stress induced by infection with *P. falciparum* [41].

Since GLN is hydrophilic and cannot directly traverse the plasma membrane, specific transmembrane transporters are required to bring GLN into and out of cells. The GLN transporters are part of a larger class of solute carriers (SLCs) and are composed of four families with different members within each: SLC1 (SLC1A5), SLC6 (SLC6A14 and SLC6A19), SLC7 (SLC7A5, SLC7A6, SLC7A7, SLC7A8, and SLC7A9), and SLC38 (SLC38A1, SLC38A2, SLC38A3, SLC38A4, SLC38A5, SLC38A6, SLC38A7, SLC38A8, SLC38A9, and SLC38A10) [42,43,44,45,46,47]. GLN transporter expression is regulated by transcription factors that respond to cellular and environmental cues [48,49,50]. For example, NF-κB can up-regulate the expression of GLN transporter transcripts in response to inflammatory signals, while other factors [e.g., hypoxia-inducible factor 1-alpha (HIF-1α) and HIF-2α] up-regulate GLN transporter genes under hypoxic conditions to correct cellular metabolism [48,49,50]. In addition, the availability of GLN in the context of the cellular metabolic state influences GLN transporter expression. In pathophysiological conditions when GLN is scarce (e.g., cellular and oxidative stress), GLN transporters are up-regulated; conversely, GLN abundance signals the down-regulation of GLN transporters to avoid excessive uptake [51]. Although changes in GLN transporters have been implicated in the pathogenesis of tuberculosis and sepsis [52,53], they remain largely unexplored in human malaria. The availability of GLN is also regulated by glutamine synthetase (GLUL), which catalyzes the ATP-dependent condensation of glutamate with ammonia to produce GLN [54]. Glutaminase 1 (GLS1) and GLS2 can also affect GLN availability, which catalyzes the conversion of GLN to glutamate and ammonia [55]. While we demonstrated that leukocytic HSP70 levels were affected by the low levels of circulating GLN in SMA, it remains unclear whether the mRNA levels of glutamine transporters or GLN metabolizing enzymes are changed in SMA.

Our recent study in 57 Kenyan children employed an unbiased approach using next-generation RNA sequencing (RNA-seq) to profile the entire expressed whole blood transcriptome in a pediatric cohort of Kenyan children with non-SMA (n = 39) or SMA (n = 18) without sickle cell anemia (SCA, HbSS genotype) [56]. The findings revealed that activating gene networks in response to hypoxic conditions is a central theme of SMA pathogenesis. Here, we extend those findings to 66 children and include children with SCA since such individuals represent the natural demographic of severe malaria, especially in holoendemic *P. falciparum* transmission regions. Results presented here focus specifically on the host immune response networks in children with non-SMA (n = 41, HbSS = 2) and SMA (n = 25, HbSS = 7). The rationale for the current study is that immune response pathways are central molecular networks that influence the development of SMA. We hypothesized that immune response pathways play a pivotal role in the pathogenesis of SMA in children with and without SCA. The investigation revealed, for the first time, that the HSP60-HSP70/TLR signaling pathway emerged as one of the top-ranked immune response pathway maps in SMA. Since we previously showed that reduced GLN is a significant predictor of SMA and an essential signal for HSP [12], GLN transporter genes and GLN metabolizing enzymes were also explored.

## 2. Materials and Methods

### 2.1. Study Design and Participants

**Figure 1:**
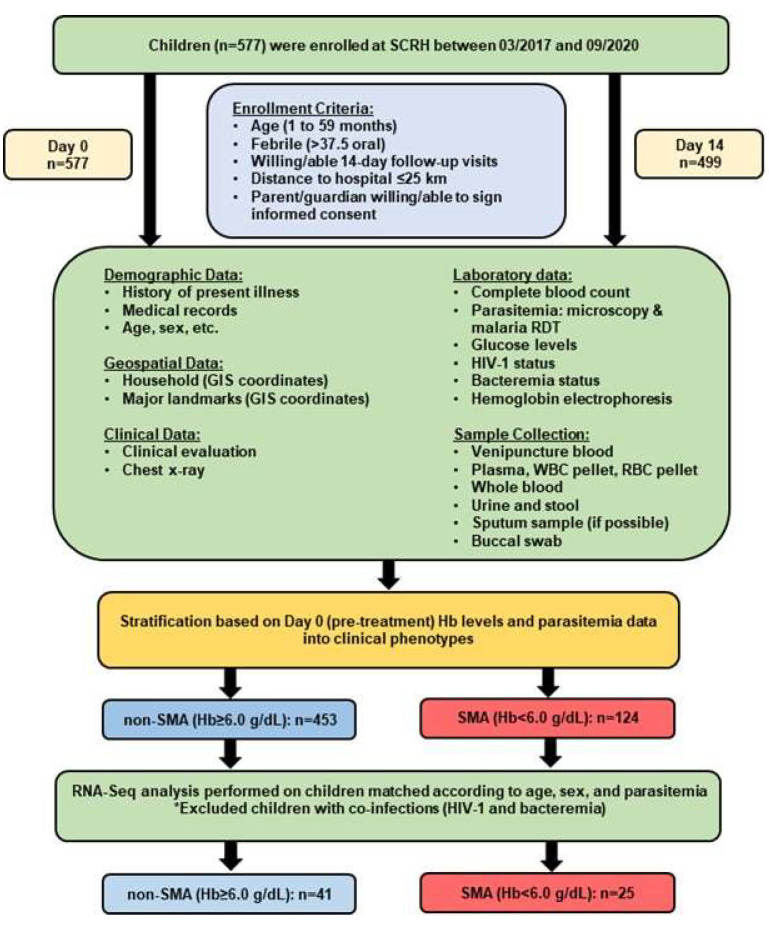
**Overall study design, data collection, and sampling strategy.** The short-term (14-day) study at Siaya County Referral Hospital (SCRH) involved children admitted with acute febrile illness. A total of 577 children (ages 1–59 months) were enrolled between March 2017 and September 2020, with 499 completing well visits. On the enrollment day (Day 0), data on demographics, geospatial information, clinical status, and laboratory results were collected. Before administering antimalarials or other medications, venipuncture blood samples (3–4 mL) were taken for laboratory analysis. Parents/guardians were asked to return their child for a well visit on day 14. Children were stratified into clinical phenotypes based on hemoglobin (Hb) levels and malaria parasitemia: non-SMA (Hb ≥ 6.0 g/dL, n = 453) and SMA (Hb < 6.0 g/dL, n = 124). For RNA-Seq analysis, children were matched by age, sex, and parasitemia, excluding those with co-infections (e.g., HIV-1 and bacteremia). A total of sixty-six children were selected for RNA-Seq: non-SMA (n = 41) and SMA (n = 25).

The prospective study was conducted (March 2017 to September 2020) at Siaya County Referral Hospital (SCRH) in western Kenya, a holoendemic *P. falciparum* malaria transmission region with high rates of malaria-related morbidity and mortality in children aged <5 years [57,58,59,60]. Female and male (sex at birth) febrile children (≥37.5 °C axillary, n = 577, age 1–59 mos.) presenting at SCRH were enrolled in the study (Figure 1).

Inclusion criteria included: *P. falciparum* parasitemia (any density), age < 5 years, distance to hospital ≤ 25 km, written informed consent from parent/guardian, intention to attend follow-up visit on day 14 (well-visit). Children were excluded if they had been previously hospitalized for any reason, had an episode of malaria within the past month, or had clinical signs consistent with cerebral malaria (rare in this setting). Written informed consent was obtained from every pediatric study participant’s parent or legal guardian during enrollment. Since severe malaria in western Kenya primarily manifests as severe malarial anemia (SMA, Hb < 6.0 g/dL, and any parasite density) [2,56,61], children were stratified into two groups: Hb ≥ 6.0 g/dL and Hb < 6.0 g/dL. Demographic and clinical data were collected at enrollment, and a physical examination was performed. Prior to treatment with antimalarials or other medications, venipuncture blood samples (3–4 mL) were collected for laboratory measures. Pre- and post-HIV test counseling was provided to the parents/guardians of all participants. All patients were treated per the Ministry of Health-Kenya guidelines. The study was approved by the Institutional Review Board of the University of New Mexico, USA (16-284), and the Maseno University Scientific and Ethics Review Committee, Kenya (MUSERC; MSU/DRPI/MUERC/00510/18).

### 2.2. Clinical Laboratory Procedures

Venipuncture peripheral blood (3–4 mL) was obtained from each study participant at enrollment (day 0) before antimalarial treatment for laboratory tests. Complete blood counts (CBC) were assessed using the Beckman Coulter ACT diff2™ (Beckman-Coulter Corporation, Miami, FL, USA). Giemsa-stained thick and thin blood smears were examined under 100× oil immersion microscopy to determine the presence/absence and species of *Plasmodium* parasites and to count the number of *P. falciparum* parasites per 300 leukocytes for estimating the parasite density based on the number of asexual malaria parasites [57]. To determine additional common causes of severe anemia in the study area [62,63], HIV-1 status, bacteremia, and HbAS status were determined. In brief, HIV-1 exposure was determined serologically using Unigold^™^ and Determine^™^ tests, while definitive HIV-1 infection was determined by pro-viral DNA PCR testing (2 separate measures 3 mos. apart) [62]. Bacteremia was evaluated by inoculating ~1.0 mL of venipuncture blood into an automated BACTEC 9050 system (Becton-Dickinson, Franklin Lakes, NJ, USA). Positive alerts were then examined by Gram staining and sub-cultured on blood agar, chocolate agar, or MacConkey agar plate (Pittsburgh, PA, USA) [63]. The presence of the HbAS trait was determined by cellulose acetate electrophoresis (Helena laboratories, Beaumont, TX, USA). An aliquot of peripheral blood (~500 μL) was mixed with an equal volume of Trizol^®^ Reagent (Thermo Fisher Scientific Inc., Waltham, MA, USA) and stored at −80 °C. Plasma from another aliquot of peripheral blood (~500 μL) was aliquoted and stored at −80 °C for later use of proteomic analysis.

### 2.3. Rna Isolation, Quantification, and Qualification

For the transcriptomic experiments, children (n = 577) were matched according to age, sex, and peripheral malaria parasitemia, excluding positive cases of HIV-1 and bacteremia. This yielded 66 individuals who were selected for RNA sequencing (RNA-Seq) studies: non-SMA (n = 41) and SMA (n = 25). Total RNA was isolated from Trizol^®^ preserved whole blood (Carlsbad, CA, USA) (500 µL) using E.Z.N.A.^®^ Total RNA Kit I (Omega Bio-Tek Inc., Norcross, GA, USA) and treated with RNase-free DNase I (New England Biolabs, Ipswich, MA, USA) to remove any contaminating DNA. Total RNA was cleaned using the RNA Clean and Concentrator Kit (ZYMO Research Corp., Tustin, CA, USA). RNA quantity was measured using a NanoDrop 2000 spectrophotometer (Thermo Fisher Scientific, Waltham, MA, USA), while the quantity and integrity of the RNA were evaluated using an Agilent 2100 Bioanalyzer (Agilent Technologies, Santa Clara, CA, USA). 

### 2.4. RNA-Sequencing and Library Construction

A total of 1 μg RNA with RNA integrity number (RIN) >8, post-globin mRNA depletion step, was used as input material for library construction. Following the manufacturer’s recommendations, the sequencing libraries were generated using NEBNext^®^ UltraTM RNA Library Prep Kit for Illumina^®^ (New England Biolabs, Ipswich, MA, USA). Briefly, mRNA enrichment was performed using poly-T oligo-attached magnetic beads. Fragmentation was performed using divalent cations under elevated temperature in NEBNext First Strand Synthesis Reaction Buffer (5X). First-strand cDNA was synthesized using a random hexamer primer and M-MuLV reverse transcriptase (RNase H-). Second-strand cDNA synthesis was subsequently performed using DNA polymerase I and RNase H. Each cDNA was ligated to a NEBNext adaptor, followed by PCR enrichment of adaptor-ligated DNA. The PCR products were then purified, and the sequencing library quality was assessed using an Agilent 2100 Bioanalyzer (Santa Clara, CA, USA). 

### 2.5. Clustering, Sequencing, and Quality Control

The clustering of the index-coded samples was performed on a cBot Cluster Generation System using PE Cluster Kit cBot-HS (Illumina^®^ Inc., San Diego, CA, USA), according to the manufacturer’s instructions. Paired-end sequencing of library preparations was performed using the Illumina^®^ platform to a depth of >20 million high-quality mappable reads. Raw reads of FASTQ format were first processed through fastp to obtain clean reads by trimming reads containing adapter and poly-N sequences and low quality. All the downstream analyses were based on clean reads.

### 2.6. Data Analysis of Study Participants’ Characteristics

Participants’ demographic, clinical, and laboratory characteristics at enrollment were analyzed using SPSS^®^ v23.0 (IBM SPSS Inc., Chicago, IL, USA). Data across the study groups was compared using Fisher’s exact and Mann–Whitney U tests. Statistical significance was set at *p* ≤ 0.050. Bivariate logistic regression analysis was conducted to identify risk factors for SMA using SPSS^®^ v23.0 (IBM SPSS Inc., Chicago, IL, USA). Variables with a *p* < 0.20 from the univariate analyses were included in the models to assess their individual associations with SMA. Odds ratios (OR) and 95% confidence intervals (CI) were calculated for each variable to evaluate the strength and direction of the associations. The significance level was set at *p* ≤ 0.050 without adjustments for multiple testing based on the sample size. Assumptions of logistic regression, including linearity of continuous variables with the logit, absence of multicollinearity, and independence of observations, were assessed to ensure the robustness of the results.

### 2.7. Mapping the Reference Genome and Quantification

The sequence reads were mapped to the reference human genome (GRCh38.p13) (NCBI/UCSC/Ensembl). Paired-end clean reads were aligned to the reference genome using the Spliced Transcripts Alignment to a Reference (STAR) software version 2.5 (Illumina^®^ Inc.). HTSeq v0.6.1 was used to count the reads mapped to each gene [64].

### 2.8. Differential Gene Expression Analysis and Visualization of Gene Expression Patterns 

For each sequenced library, the read counts were converted to fragments per kilobase of transcript per million mapped reads (FPKMs) based on the length of the gene. Differential gene expression analysis was then performed using edgeR (3.16.5) [65]. False discovery rate (FDR) adjusted *p*-value (*P*adj) was calculated using the Benjamini–Hochberg method [66], and *P*adj of 0.050 was set as the threshold for significance. To visualize the gene expression patterns, next-generation clustered heatmaps (NG-CHMs) of DEGs were generated using hierarchical clustering with Euclidean and Ward’s distance and clustering methods [67].

### 2.9. Leukocytic Immune Cell Profiling

The relative percentage of different immune cell types/subtypes in peripheral blood was calculated using CIBERSORTx [68,69], which processes gene expression data from a bulk admixture of various cell types to estimate the abundance of each cell type in the sample [68]. We used the curated signature matrix file, LM22, as the reference to deconvolute the relative fraction of different cell types in whole blood, identifying 22 types/subtypes of leukocytes. Cell type-specific gene expression levels were imputed at the sample level, with the output presenting as the fractional proportions in whole blood for each study participant. A two-sample *t*-test with Welch’s correction was used to determine whether the relative proportions (%) of immune cell types, presented as mean (SEM), differed between the non-SMA (n = 41) and SMA (n = 25) groups. GraphPad Prism v9.5.1. (GraphPad Software, Boston, MA, USA) was employed to generate a heatmap and relative proportions (%) of the 22 leukocyte cell types/subtypes at the individual patient level for both non-SMA and SMA groups.

### 2.10. Weighted Gene Co-Expression Network Analysis

Weighted gene co-expression network analysis (WGCNA) was used to identify gene modules associated with traits of interest using the WGCNA package in R (version 1.72-5) [70]. A soft-thresholding power of 4 was selected based on the scale-free topology criterion, optimizing for an R^2^ > 0.8. The resulting adjacency matrix was transformed into a topological overlap matrix (TOM) to assess gene interconnectedness. Modules were identified via hierarchical clustering with a dynamic tree cut, using a minimum module size of 500 genes. Module eigengenes were correlated with clinical traits (non-SMA and SMA) using Pearson correlation, with *p*-values adjusted for multiple testing by the Benjamini–Hochberg method. As all genes were analyzed in a single block, the merging of correlated eigen genes was not performed. Functional enrichment analysis was conducted with the gprofiler2 package in R (version 0.2.3), focusing on GO terms and Reactome pathways, with significance assessed using hypergeometric tests and *p*-values adjusted for multiple comparisons. 

### 2.11. Pathway Enrichment Analysis

To identify significant gene pathways and network processes in SMA, pathway enrichment analysis was performed using the Metacore^TM^ pathway analysis software suite (v2024, Clarivate Analytics, Philadelphia, PA, USA, https://clarivate.com/products/metacore/, accessed on 18 September 2024).

### 2.12. Validation of Transcriptome Profiles

An independent cohort of 50 children (non-SMA, n = 38; SMA, n = 12) was used to validate our findings. RNA was extracted from the peripheral blood of the selected children using the RNeasy Mini Kit (Qiagen, Germantown, MD, USA). RNA was amplified and biotinylated using the Illumina^®^ TotalPrep RNA Amplification Kit (Thermo Fisher Scientific) and quantified on an Agilent 2100 Bioanalyser. Specifically, 750 ng cRNA per sample was hybridized to Illumina^®^ human HT-12 v4 expression BeadChips harboring 47,231 probes (Illumina^®^ Inc.) and scanned with a BeadStation 500GX (Illumina^®^ Inc.) per the manufacturer’s recommendations (Illumina^®^ Inc.). Illumina^®^s’ BeadStudio software version 3.2 was utilized to filter the data prior to normalization. Illumina^®^ probe profile expression data were normalized using quantile normalization and corrected for batch processing effects [71]. DEGs between SMA and non-SMA were identified using significance analysis of microarrays (SAM) [72]. GraphPad Prism v9.5.1. (GraphPad Software) was employed to generate a heatmap and a scatter plot of the expression patterns of common genes in both the Novogene and Illumina^®^ hHT-12 datasets.

### 2.13. Proteomic Validation on the Alteration of HSP60-HSP70-TLR2/4 Signaling Pathway in SMA

We selected 40 children (SMA, n = 18, as cases; non-SMA, n = 22, as controls) whose whole blood was used for transcriptomic profiling (described above) to conduct proteomic analysis on their plasma samples. Plasma was obtained by centrifuging fresh venipuncture peripheral blood samples at 1000× *g* for 10 min at ambient temperature, followed by transferring the top aqueous phase to a fresh tube and then stored in aliquots at −80 °C until use. All plasma samples selected for use had sufficient volumes and quality and underwent no previous freeze-thaw cycles before assaying on the 7k SomaScan Assay v4.1 platform (SomaLogic), following the manufacturer’s protocol. Briefly, plasma samples were diluted and incubated with dilution-specific SOMAmers, pre-synthesized with a fluorophore, photocleavable linker, and biotin. Plasma proteins bound to a biotin-tagged SOMAmer were attached to streptavidin magnet beads and thus retained as part of a pellet in a magnet field, while the unbound proteins remained in solution and were washed away. The photocleavable linker was dissociated by exposure to ultraviolet (UV) light, releasing protein-SOMAmer complexes into the solution. The SOMAmers were released by denaturing the proteins, and fluorophores were measured on a microarray chip. The fluorescence intensity, measured in relative fluorescence units (RFU), inferred the quantity of epitope in the original plasma sample [73]. The aptamer-based scan had a median limit of detection (LOD) of 125 fM, or 5.3 pg/mL for a protein/peptide [74].

Proteomic data processing and analysis were conducted as follows: Data were standardized using a sample-by-sample adjustment to overall signals within the plasma dilutions, while calibration constituted an overall plate and SOMAmer-by-SOMAmer adjustments to decrease between-plate variability. The final analysis incorporated 35 samples that passed the quality control check. Protein measurements (RFUs) were compared between non-SMA and SMA groups using a generalized linear model with a negative binomial distribution. The proteins were matched to their respective transcripts using network algorithms in MetaCore^TM^ (v2024, Clarivate Analytics, Philadelphia, PA, USA, https://clarivate.com/products/metacore/, accessed on 9 September 2024), and correlation analyses were determined using Spearman’s test. Significant (*p* ≤ 0.050) transcripts and proteins were compared. GraphPad Prism v9.5.1. (GraphPad Software) was used to create a heatmap and a scatter plot of the expression patterns of common genes in both the Novogene and proteomics datasets.

## 3. Results

### 3.1. Demographic, Clinical, and Laboratory Characteristics of the Study Participants

Age- and sex-matched children without co-infections (i.e., HIV-1 and bacteremia) with peripheral *P. falciparum* parasitemia (any density) were stratified into two groups based on Hb levels: Hb ≥ 6.0 g/dL (non-SMA, n = 41) and Hb < 6.0 g/dL (SMA, n = 25). The overall study design is shown in Figure S1, and the study participants’ demographic, clinical, and laboratory characteristics upon presentation at the hospital (day 0, pre-treatment) are shown in Table 1.

Children with SMA had lower axillary temperatures (*p* = 0.051) and comparable glucose levels (*p* = 0.967). Consistent with the *a priori* classification based on Hb concentrations, children with SMA had lower hematocrit levels (*p* = 1.242 × 10^−11^) and RBCs (*p* = 1.790 × 10^−11^). Conversely, SMA was characterized by elevated red blood cell distribution width (*p* = 4.050 × 10^−4^), mean corpuscular volume (*p* = 0.002), and mean corpuscular hemoglobin concentrations (*p* = 0.022). Elevations in the SMA group were also witnessed for white blood cells (*p* = 1.393 × 10^−4^), lymphocytes (*p* = 5.509 × 10^−6^), and monocytes (*p* = 0.022). Comparable levels for additional hematological variables were observed: platelet (*p* = 0.615), neutrophil (*p* = 0.438), and granulocyte (*p* = 0.373) counts. Parasitological indices (i.e., parasite density and stratified levels) did not significantly vary across the two groups (*p* = 0.155 and *p* = 0.134, respectively). However, the distribution of sickle-cell trait status differed between the groups, characterized by a lower proportion of HbAA and HbAS and a higher proportion of HbSS in SMA (*p* = 0.029).

### 3.2. Demographic, Clinical, and Laboratory Predictors of Severe Malarial Anemia

**Figure 2:**
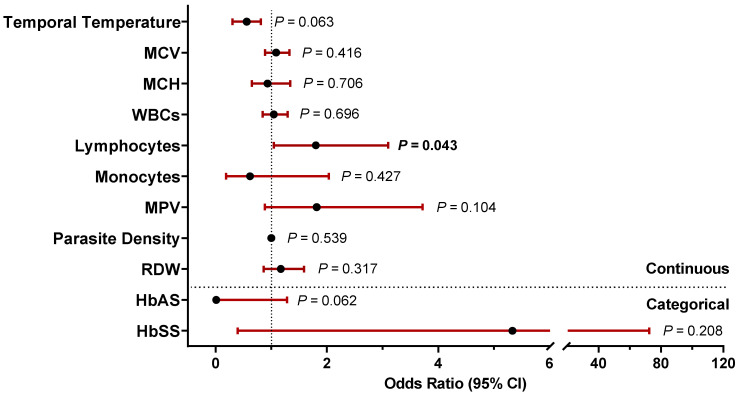
**Predictors of malarial anemia severity.** Data are presented as odds ratios (ORs) with 95% confidence intervals (CI) determined using a bivariate logistic regression model with patient’s with at *p* < 0.200 included as predictors. Black dots represent the ORs, while the red lines represent 95% CI. Abbreviations: MCV (mean corpuscular volume), MCH (mean corpuscular hemoglobin), WBCs (white blood cells), MPV (mean platelet volume), RDW (red blood cell distribution width), HbAS (hemoglobin AS), and HbSS (hemoglobin SS). *p*-values ≤ 0.050 were considered significant and are indicated in bold.

Logistic regression modeling was utilized to determine predictors of SMA by including all variables with a *p* < 0.200 in the univariate analysis (Figure 2). An elevated temporal temperature on admission was associated with a reduced risk of SMA [OR = 0.440 (95%CI = 0.185–1.0460) *p* = 0.063]. Elevated lymphocyte counts also increased the risk of SMA [OR = 1.800 (95%CI = 1.045–3.101) *p* = 0.034]. Carriage of HbAS was protective against SMA [OR = 0.007 (95%CI < 0.001–1.281) *p* = 0.062], while inheritance of HbSS markedly increased susceptibility to SMA [OR = 5.333 (95%CI = 0.393–72.403) *p* = 0.208]. Despite the limited sample size, elevated lymphocytes emerged as a significant predictor of SMA. Additional patient characteristics showed trends towards either protection (i.e., elevated temperature and HbAS) or enhanced risk of SMA (i.e., HbSS).

### 3.3. Differential Gene Expression Analysis Uncovers a Significantly Altered Transcriptomic Landscape in Severe Malarial Anemia

**Figure 3:**
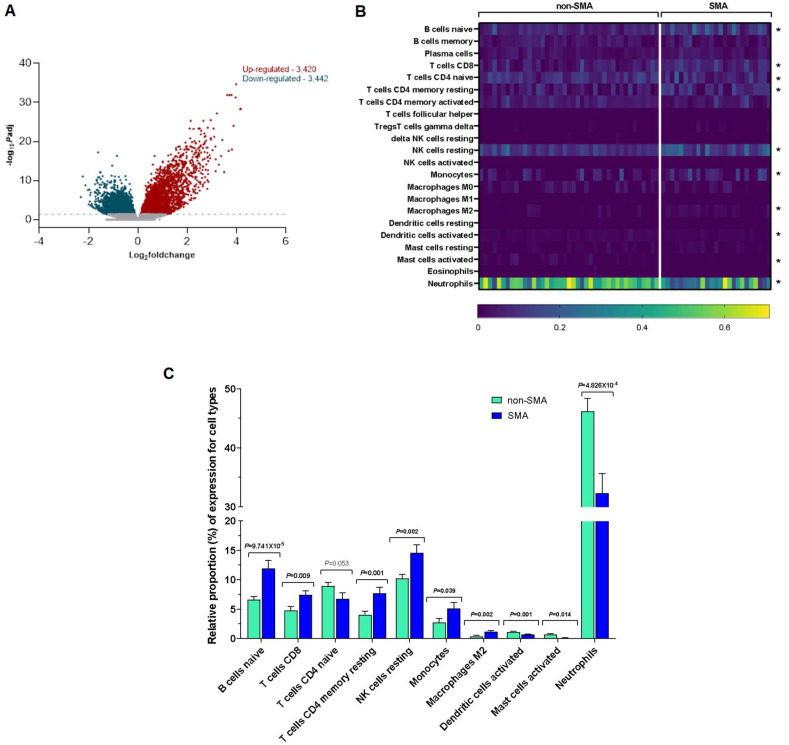
**Differential gene expression analysis and leukocytic immune profiling in children with severe malarial anemia.** (**A**) The volcano plot shows 3420 up-regulated (red) and 3442 down-regulated (turquoise) genes in children with SMA. The horizontal axis shows the Log_2_foldchange, while the vertical axis shows the −log_10_ *P*adj values. The horizontal dotted line corresponds to 1.301 (i.e., −log_10_ *P*adj significance threshold at 0.050). (**B**) The composition of different blood cell types was analyzed using CIBERSORTx for deconvolution. The LM22 signature matrix file was used to estimate cellular frequencies. A heatmap illustrates the expression levels of 22 leukocyte cell types/subtypes at the individual patient level for both non-SMA (Hb ≥ 6.0 g/dL, n = 41) and SMA (Hb < 6.0 g/dL, n = 25) groups. Significant differences in immune cell proportions between the groups are marked with an asterisk (*), determined by Welch-corrected, two-sided, two-sample *t*-tests (*p* ≤ 0.050). (**C**) The relative proportions (%) of immune cell types that differ between the non-SMA (n = 41) and SMA (n = 25) groups are shown as mean (SEM), based on bivariate analysis using two-sided, two-sample *t*-tests with Welch correction. Gray font indicated borderline significance.

RNA-seq of the entire expressed transcriptome in whole blood revealed 6862 differentially expressed genes (DEGs) in SMA relative to non-SMA after correcting for the false discovery rate (FDR, *P*adj < 0.050). Of these DEGs, 3420 genes exhibited up-regulation, while 3442 genes displayed down-regulation (Figure 3A), suggesting a complex transcriptional response associated with SMA pathogenesis.

### 3.4. Altered Leukocytic Immune Cell Profiles in Severe Malarial Anemia

A bioinformatic analysis using CIBERSORTx was conducted to investigate the leukocytic immune profiles in children who develop SMA. Despite individual variability, the heatmap analysis demonstrated that ten immune cell types were differentially expressed with a significance level of *p* ≤ 0.050 (Figure 3B). Children with SMA showed elevated levels of naïve B cells (*p* = 9.741 × 10^−5^), CD8 T cells (*p* = 0.009), CD4 memory resting T cells (*p* = 0.001), resting NK cells (*p* = 0.002), monocytes (*p* = 0.039), and M2 macrophages (*p* = 0.002) (Figure 3C). Conversely, there was a notable decrease in the proportion of activated dendritic cells (*p* = 0.001), activated mast cells (*p* = 0.014), and neutrophils (*p* = 4.826 × 10^−4^), with a slight reduction in naïve CD4 T cells (*p* = 0.053) in the SMA group (Figure 3C). These patterns suggest that children with SMA experience a diminished antigenic response, lowered immune priming, and a shift toward cellular proliferation and tissue repair.

### 3.5. Co-Expression Network Analysis Reveals Distinct Gene Modules Associated with Non-SMA and SMA Phenotypes

**Figure 4:**
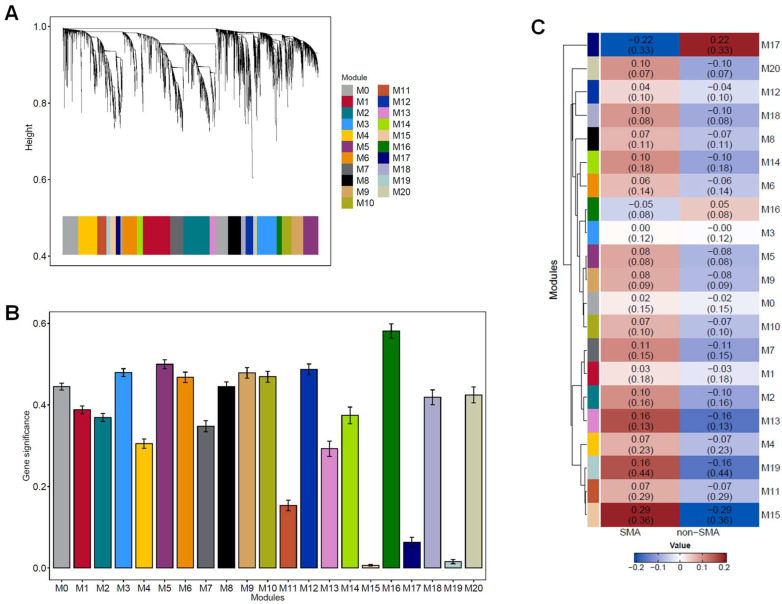
**WGCNA modules of co-expressed genes.** (**A**) WGCNA cluster dendrogram obtained by hierarchical clustering performed using the TOM-based dissimilarity matrix (1—TOM) as the distance measure. A total of 21 gene co-expression network modules (M0–M20) were identified using WGCNA (single block analysis). The branches refer to clusters of genes that are highly connected, and each vertical line represents a single gene. The colors in the horizontal bar represent the 21 gene co-expression modules. (**B**) Module significance values of the identified modules associated with clinical traits (non-SMA and SMA). Module significance value indicates the summary (mean) of gene significance of all genes in each module, with column colors indicating different modules. (**C**) Heatmap of module-clinical trait relationships. The heatmap shows the correlation between WGCNA module eigengenes and clinical status. Each cell contains the correlation coefficient and standard deviation in parenthesis. Rows and columns represent modules and clinical features, respectively. The color intensity represents the strength of the correlation. Red indicates a positive correlation, while blue represents a negative correlation.

WGCNA was utilized to identify modules of co-expressed genes in non-SMA and SMA groups. Hierarchical clustering on the TOM-based dissimilarity identified modules with a dynamic tree cut and a minimum module size of 500 genes, resulting in 21 modules (M, Figure 4A). Module eigengenes were correlated with clinical traits (non-SMA and SMA) to explore relationships between gene expression and clinical outcomes, revealing significance for M15 (*p* = 0.006) and M19 (*p* = 0.016, Figure 4B). The heatmap showing the mean gene correlation for each module is shown in Figure 4C.

**Figure 5:**
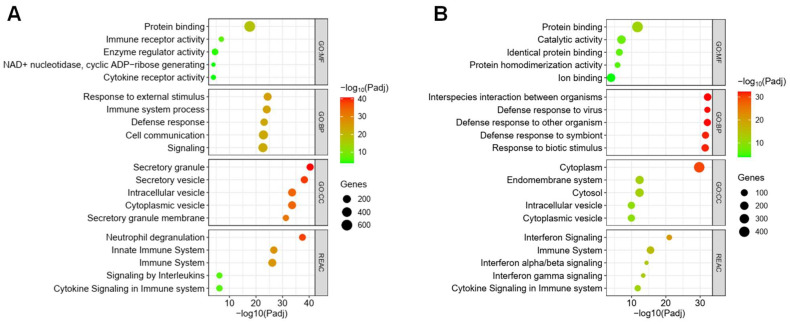
**Functional enrichment analysis of M15 and M19**. Functional enrichment plots illustrate biological pathways and processes associated with the co-expressed genes in the WGCNA modules. (**A**) Enrichment analysis for M15. (**B**) Enrichment analysis for M19. The *x*-axis represents the −log10 of the FDR-adjusted *p*-value, indicating the significance of enrichment, while the *y*-axis shows the enriched terms. The top five most significant terms are displayed for each module across different enrichment categories: GO (gene ontology), MF (molecular function), CC (cellular component), BP (biological process), and REAC (Reactome pathways).

Based on their significance values, functional enrichment analyses were performed for M15 (804 genes) and M19 (624 genes). The top five GO molecular functions, biological processes, cellular components, and Reactome output for M15 and M19 are presented in Figure 5A and Figure 5B, respectively. The integrated summary of the enrichment analysis for M15 illustrates the central role of the module in immune regulation, signaling, and cellular responses, specifically through pathways involved in neutrophil degranulation, innate immune responses, and TLR cascades. The amalgamated information from M19 reveals a substantial involvement of the identified gene set in immune response mechanisms related to defense, protein interactions, and cytokine signaling.

### 3.6. Severe Malarial Anemia Is Characterized by Immune Dysregulation in the HSP60-HSP70-TLR2/4 Signaling Pathway

**Figure 6:**
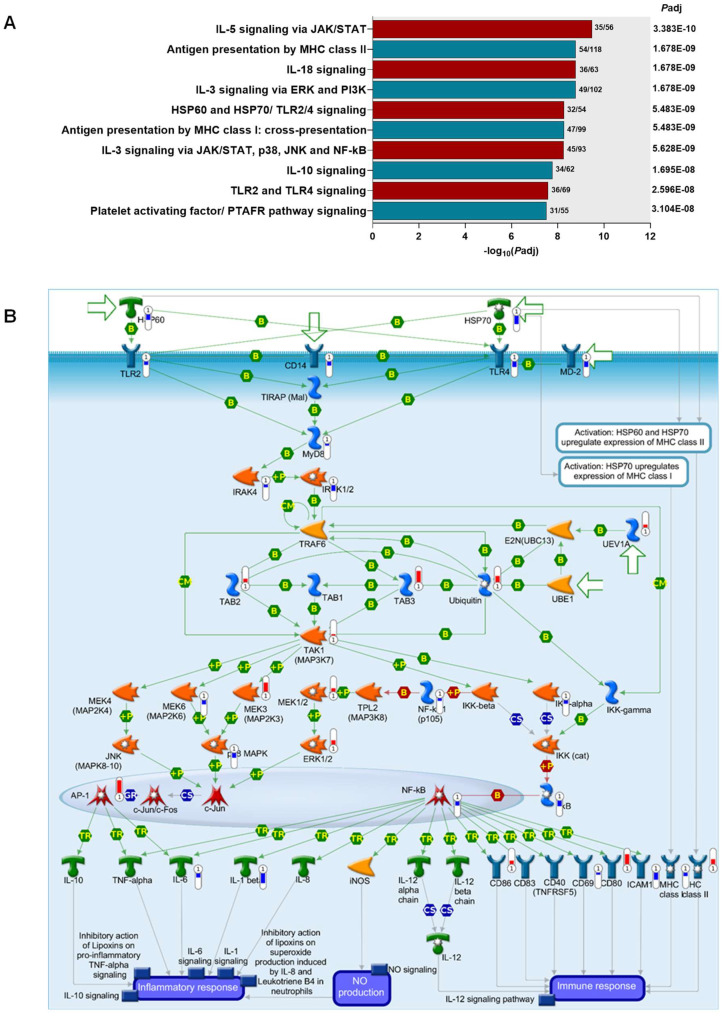
**Top emergent immune response canonical pathway maps in children with severe malarial anemia.** (**A**) The top 10-ranked immune response canonical pathway maps were generated using MetaCore^TM^ from the RNA-seq analysis in non-SMA (Hb ≥ 6.0 g/dL, n = 41) versus SMA (Hb < 6.0 g/dL, n = 25) at *P*adj < 0.05. Protein or protein complexes are shown as individual symbols. The left *Y*-axis indicates the biological pathways for human metabolism and cell signaling in immune response networks. The number of DEGs in the dataset is shown relative to the total number in the pathway. The right *Y*-axis shows *P*adj values for each pathway map. The *X*-axis represents the −log_10_(*P*adj) values. (**B**) The HSP60-HSP70-TLR2/4 signaling pathway (*P*adj = 5.483 × 10^−9^) with the *P*adj DEGs representing 32/54 nodes. The transcripts are the thermometers shown in red (up-regulated) or blue (down-regulated). The details of symbols used in these figures are available at the following site: https://portal.genego.com/legends/MetaCoreQuickReferenceGuide.pdf (accessed on 20 September 2024). See also Figure S1.

Given that the immune response emerged as a central feature in the WGCNA, canonical pathway maps for the DEGs (*P*adj < 0.050) were generated using Metacore^TM^, selectively filtered to identify networks associated with immune activity. The top 10 emergent immune response pathways for the DEGs included a signaling network enriched for HSPs and TLRs, i.e., the HSP60-HSP70-TLR2/4 signaling pathway (*P*adj = 5.483 × 10^−9^, 3rd ranked according to *p*-value, Figure 6A). Based on our previous findings on HSPs in childhood malaria [12], we expanded these investigations and focused on this immune response pathway. Children with SMA had significant dysregulation for 32 out of 54 nodes in the HSP60-HSP70-TLR2/4 signaling pathway (Figure 6B). The 32 mapped nodes in the pathway comprise 47 genes, of which 17 were up-regulated and 30 were down-regulated. The Log_2_foldchange (log_2_FC) and *P*adj for each of the genes representing the proteins are shown in Table S1. To gain an improved understanding of the underlying molecular mechanisms that influence disease severity, the known signaling actions of genes within the HSP60-HSP70-TLR2/4 pathway are described below in the context of actual (observed) expression changes (i.e., up- or down-regulation) in children with SMA (Figure 6B).

Of relevance to the role of HSPs in human malaria pathogenesis, transcripts for HSP60 (*HSPD1*) were down-regulated in SMA (log_2_FC = −0.81), as were HSP70 family members: *HSPA1A* (−1.31), *HSPA1B* (−0.99), *HSPA4* (−0.33), *HSPA4L* (−0.68), *HSPA5* (−0.51), and *HSPA6* (−0.81). Although increased HSP60 and HSP70 levels enhance the surface expression of MHC class II, despite their reduction, the following class II transcripts were still up-regulated: HLA-DMA (+0.38), HLA-DOA (+0.70), HLA-DOB (+0.42), HLA-DPB1 (+0.56), and HLA-DPB2 (+0.75). However, consistent with the known action of HSP70 increasing MHC class I expression, reduced HSP70 transcripts paralleled decreased class I expression for B2M (−0.37), HLA-B (−0.33), HLA-C (−0.54), and HLA-E (−0.39).

Down-regulation of gene expression was also observed for TLR2 (−0.53) and TLR4 (−0.83). HSP60 and HSP70 can bind to TLR2 and TLR4, forming a complex with CD14 (−0.60). For TLR4-mediated responses to HSP60 and HSP70, MD-2 (−0.89) is necessary. TLR2 and TLR4 then bind to TIRAP (Mal) for recruitment of MyD88 (−0.32), which binds to IRAK4 (−0.36), subsequently phosphorylating/activating IRAK1 (+0.23) and IRAK2 (−0.81). Thus, the observed reduction in the signaling molecules in children with SMA elicits the expected known actions, except for the up-regulation present for IRAK1. However, since IRAK1 and IRAK2 form a complex, the higher down-regulation in IRAK2 likely overrides the moderate increase in IRAK1.

In addition, UEV1A (+0.45) undergoes autoactivation, as well as activation of TAB2 (+0.57) and TAB3 (+1.82) by ubiquitination [Ubiquitin B (UBB), +0.94]. TAB2/3 can form a complex with TAK1 (MAP3K7, +0.18) for activation of IKK-alpha (−0.49), which, once phosphorylated in the IKK complex, can degrade I-kB (−0.91), resulting in translocation of NF-kB1 (−0.40) into the nucleus for subsequent activation of gene expression of IL-6 (−0.60), IL-1β (−1.51), ICAM1 (−1.04), CD69 (−0.29), CD80 (+1.54), and CD86 (+0.54). In children with SMA, decreased IKK-alpha transcripts appear responsible for reduced NF-kB1 transcriptional regulation of its downstream targets, except for CD80 and CD86, which were up-regulated. However, this observation may be explained by alternative signaling pathways that can activate CD80 and CD86, such as CD-40-CD40L interactions and the JAK/STAT pathway, which is not annotated in the current signaling map [75,76].

Degradation of NF-kB1 (−0.40) liberates TPL2 (MAP3K8) for activation of MEK1/2 (+0.55), followed by phosphorylation of ERK1/2 (+0.60) and the subsequent phosphorylation of c-Jun, which forms a heterodimer with c-Fos. This complex activates AP-1 [(JUNB, −0.57) and (JUND, +1.95)], which can induce IL-6 (−0.60) expression. Convergent signaling for activation of AP-1 [(JUNB, −0.57) and (JUND, +1.95)] also occurs by signaling through TAK1 (MAP3K7, +0.18), resulting in phosphorylation of MEK3 (MAP2K3, +1.44) and MEK6 (MAP2K6, −0.68), and the subsequent phosphorylation of p38 MAPK (−0.78). Thus, in children with SMA, it appears that TAK1/MEK3 could signal through the enhanced levels of AP1, but despite such, transcriptional regulation of downstream targets (e.g., IL-6) does not appear to be enhanced. In conclusion, a substantial alteration in the cellular signaling cascade impacts the function of HSPs, MHC expression, TLR signaling, and various other cellular responses (e.g., NF-kB and AP-1 signaling).

### 3.7. Unsupervised Hierarchical Clustering Analysis of Gene Expression Profiles in the HSP60-HSP70-TLR2/4 Signaling Pathway

**Figure 7:**
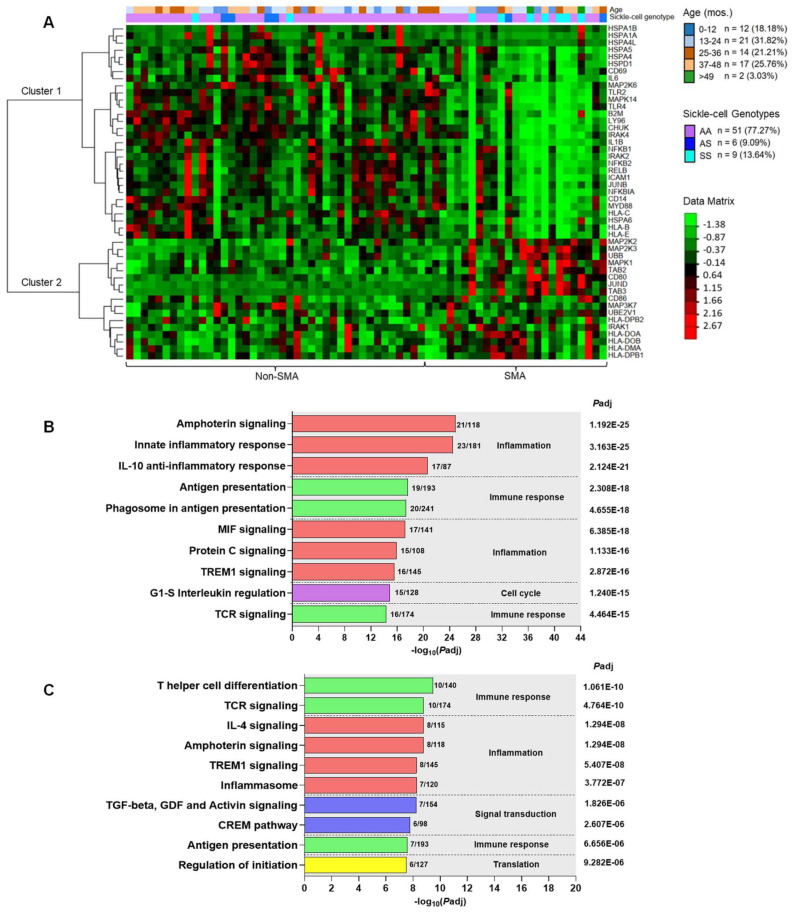
**Unsupervised hierarchical clustering of RNA-seq data showing differentially expressed genes in the HSP60-HSP70-TLR2/4 pathway**. (**A**) Heatmap showing expression values for each of the 47 DEGs selected based on *P*adj ≤ 0.050 (rows) normalized across all samples (columns). Dendrogram of hierarchical clustering of genes based on Euclidean distance of z-score data. Age distributions and sickle cell trait status are shown, along with the data matrix indicating the magnitude of up-regulated (red) and down-regulated (green) genes. (**B**) Top 10-ranked process networks generated in MetaCore^TM^ for cluster 1 (down-regulated) DEGs. (**C**) Top 10-ranked process networks generated in MetaCore^TM^ for cluster 2 (up-regulated) DEGs. Process networks are represented on the left *Y*-axis. The number of DEGs in the dataset is shown relative to the total number in the process network. The right *Y*-axis indicates the *P*adj value, and the *X*-axis shows the −log_10_(*P*adj) values.

An unsupervised hierarchical clustering heatmap was generated to visualize individual gene expression patterns for DEGs (*P*adj < 0.050) in the HSP60-HSP70-TLR2/4 signaling pathway in children with non-SMA and SMA, along with information about stratified age groups and sickle-cell genotype for each study participant (Figure 7A). This analysis revealed two distinct (major) clusters, with cluster 1 composed of 30 down-regulated genes in SMA and cluster 2 containing 17 up-regulated genes. In children with SCA, particularly those in the SMA group, there was an exacerbation of both the down- and up-regulated genes in clusters 1 and 2, respectively. To further explore the biological functions of the DEGs in clusters 1 and 2, MetaCore^TM^ was used to generate the top 10 ranked process networks. The top-ranked process networks for cluster 1 (down-regulated) included Inflammation-Amphoterin Signaling (*P*adj = 1.192061 × 10^−25^) and Inflammation-Innate Inflammatory Response (*P*adj = 3.163061 × 10^−25^, Figure 7B). Common genes in multiple pathways were TLR4 and TLR2, MyD88, IRAK1/2, IKK-alpha (I-kappa-B kinase alpha), NF-kB (various forms including p100, p105, p52, and p50), NFKBIA (I-kappa-B alpha), AP-1 (Activator Protein 1), ICAM1, and IL-1 beta (Table S2), suggesting that children with SMA have an impaired immune response, weakened TLR-signaling, and a dysregulated inflammatory balance. The top-ranked process networks for cluster 2 (up-regulated) contained Immune Response-T Helper Cell Differentiation (*P*adj = 1.061 × 10^−10^) and Immune Response-TCR signaling (*P*adj = 4.764061 × 10^−10^, Figure 7C) with an overrepresentation of up-regulation of CD86, ERK1/2 (MAPK1), MEK1/2 (MAP2K1/2), TAK1 (MAP3K7), IRAK1/2, and MHC class II (Table S2), indicating an enhanced response to specific immune and inflammatory pathways associated with differentiation and activation of T helper cells and pathways associated with T cell receptors. These findings reveal an interplay between altered HSP60 and HSP70 expression and TLR signaling in SMA, leading to compromised cellular stress responses and potential increases in damage and inflammation, suggesting compensatory and pathological activations.

### 3.8. Dysregulation of the HSP60-HSP70-TLR2/4 Signaling Pathway Is a Central Feature of Severe Malarial Anemia in Children with and without Sickle Cell Anemia

To capture the representative natural landscape of children who present at hospitals with SMA in holoendemic *P. falciparum* transmission regions, carriers of all sickle cell genotypes were included in the aforementioned transcriptomic analyses. Consistent with the expected enrichment of HbSS carriers in children with severe malaria [77], the following distribution was present in the cohort: non-SMA (HbSS carriers = 2/41, 4.9%) and SMA (HbSS carriers = 7/25, 28.0%, see Table 1). To mitigate any potential confounding effects of HbSS carriage on our findings related to immune dysregulation in the HSP60-HSP70-TLR2/4 signaling pathway, canonical pathway mapping was performed in children without SCA in the non-SMA (n = 39) and SMA (n = 18) groups. This analysis confirmed significant dysregulation of DEGs in the HSP60-HSP70-TLR2/4 signaling pathway (54/54 nodes, *P*adj = 9.041 × 10^−13^, Figure S1), confirming that dysregulation in the signaling pathway is a general feature of SMA in children with and without sickle cell anemia.

### 3.9. Validation of the HSP60-HSP70-TLR2/4 Signaling Pathway DEGs with High-Throughput Gene Expression Profiling

**Figure 8:**
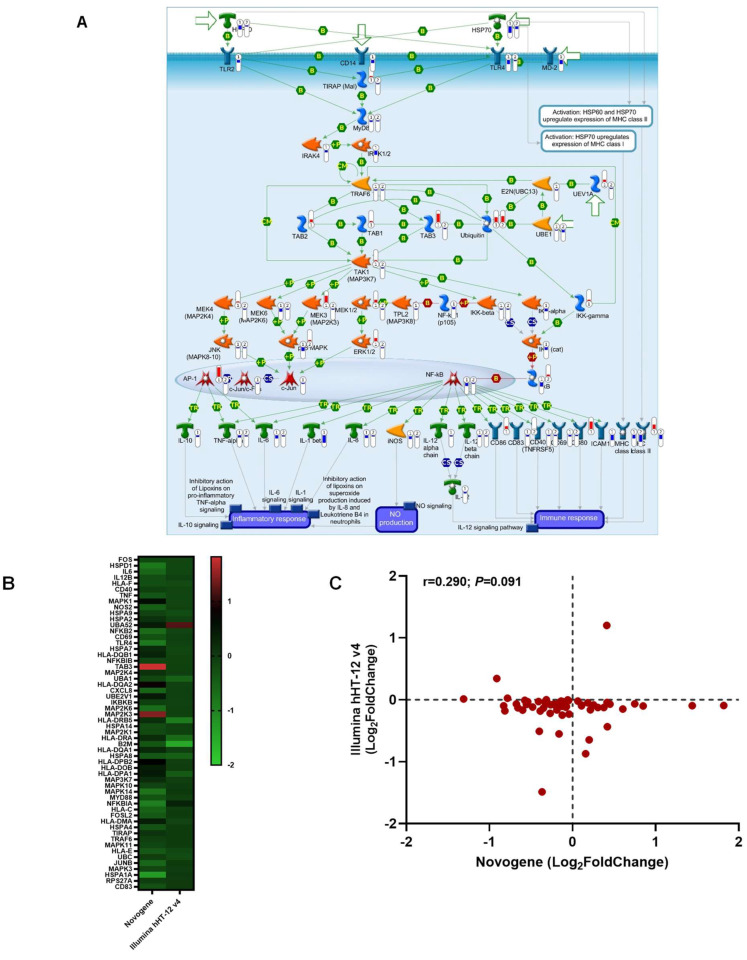
**Comparative analysis of RNA-seq and Illumina^®^ platforms for HSP60-HSP70-TLR2/4 signaling pathway.** (**A**) Gene abundance in the RNA-seq dataset (Novogene) was compared with measures obtained using the Illumina^®^ HumanHT-12 v4 beadchip covering >47,000 transcripts on the Illumina^®^ “iScanSQ” platform using MetaCore™. There were 66 children in the RNA-seq experiment (n = 41 non-SMA and n = 25 SMA) and 50 children in the Illumina^®^ platform experiment (n = 38 non-SMA and n = 12 SMA). The RNA-seq data mapped to 54/54 nodes (*P*adj = 9.041 × 10^−13^), while the Illumina^®^ platform mapped to 28/54 nodes (*P*adj = 5.452 × 10^−5^). The missing data in the Illumina^®^ set was because those genes representing the proteins were absent in the assay format. The details of symbols used in these figures are available at the following site: https://portal.genego.com/legends/MetaCoreQuickReferenceGuide.pdf (accessed on 9 September 2024) (**B**) Heatmap showing the comparison of Novogene/Illumina^®^ HumanHT-12 v4 pairs between the two datasets. The *Y*-axis depicts the matched transcript pairs, while the *X*-axis represents the assay type. The color scale depicts fold regulation (Log_2_). (**C**) Correlation scatter plot demonstrating the relationship between significantly expressed protein targets (Log_2_FoldChange; *Y*-axis) and genes (Log_2_FoldChange; *X*-axis). A two-tailed Spearman’s test indicated concordance between the transcript pairs at r  = 0.290 and *p* = 0.091.

For validation of the DEGs in the HSP60-HSP70-TLR2/4 signaling pathway, global gene expression profiling (>19,185 transcripts, Illumina^®^ HumanHT-12 v4 beadchip) was performed on whole blood samples from a separate cohort of children (recruited April 2004 to September 2015). The experiment was performed on samples collected prior to treatment interventions from 50 children representing two extremes of clinical malaria phenotypes: mild malarial anemia (non-SMA; Hb levels of 8.1–12.4 g/dL; n = 38, average Hb = 9.3 g/dL) and severe malarial anemia (SMA; Hb levels of 4.1–5.9 g/dL; n = 12, average Hb = 5.3 g/dL). Figure S2 illustrates the overall study design, while Table S3 provides the study participants’ detailed demographic, clinical, and laboratory characteristics. Comparative analysis of the RNA-seq and Illumina^®^ platforms was performed by mapping both datasets onto the HSP60-HSP70-TLR2/4 signaling pathway using MetaCore^TM^ without any thresholds to depict all the expression patterns (Figure 8A). The Illumina^®^ platform mapped to 28/54 nodes in the HSP60-HSP70-TLR2/4 signaling pathway and was highly significant (*P*adj = 5.452 × 10^−5^), confirming the findings for the RNA-seq analysis that mapped to 54/54 nodes (*P*adj = 9.041 × 10^−13^). Cluster analysis was then performed on the HSP60-HSP70-TLR2/4 signaling pathway genes in both datasets (n = 56 genes). The heatmap analysis revealed consistent fold change patterns and directionality for most genes (Figure 8B). The cross-platform comparison showed a linear relationship with a correlation coefficient of r = 0.290 and a *p*-value of 0.091 (Figure 8C). Collectively, validation with the beadchip array yielded concordance with the directionality and magnitude of the RNA-seq data captured for the HSP60-HSP70-TLR2/4 signaling pathway.

### 3.10. Integration of RNA-seq and Proteome Data for the HSP60-HSP70-TLR2/4 Signaling Pathway

**Figure 9:**
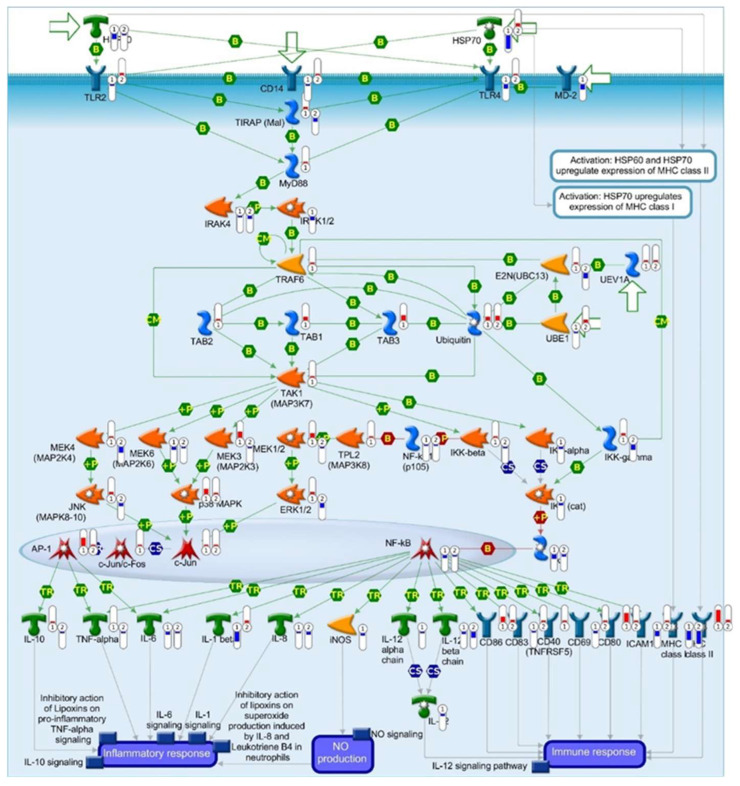
Comparative analysis of transcript and protein abundance in the HSP60-HSP70-TLR2/4 signaling pathway in children with severe malarial anemia. MetaCore™ was employed to illustrate genes with their respective protein products in a cohort of 35 children (non-SMA, n = 19; SMA, n = 16) for whom both RNA and protein data were available. Pathway maps were created without applying thresholds to view all transcript and protein relationships. The transcriptome data mapped to 54/54 nodes (*P*adj = 9.041 × 10^−13^), while the proteome data mapped to 30/54 nodes (*P*adj = 7.413 × 10^−10^), illustrating the strong biological relationship between the transcriptome and proteome data in the pathway. The lack of a protein match for some of the nodes was because 12 of the proteins were not in the SomaScan array. The details of symbols used in these figures are available at the following site: https://portal.genego.com/legends/MetaCoreQuickReferenceGuide.pdf (accessed on 17 September 2024).

To assess the relationship between DEGs in the HSP60-HSP70-TLR2/4 signaling pathway and levels of corresponding proteins, transcriptomic data from whole blood were compared with protein abundance data measured in plasma using the 7k SomaScan platform. For these experiments, 35 children [non-SMA (n = 19) and SMA (n = 16)] out of the overall cohort (n = 66) had both RNA-seq and proteomic data for comparison. Concomitant mapping of the RNA-seq and proteomic datasets to the HSP60-HSP70-TLR2/4 signaling pathway was performed with MetaCore^TM^ in the absence of thresholds to fully illustrate the maximum number of transcript/protein pairs (Figure 9). The proteomic dataset mapped to 38/54 nodes in the HSP60-HSP70-TLR2/4 signaling pathway and was highly significant (*P*adj = 7.413 × 10^−10^), while the RNA-seq data mapped to 54/54 nodes (*P*adj = 9.041 × 10^−13^). As expected for concomitant transcript and protein measurements that are frequently divergent due to temporal dynamics and post-transcriptional, translational, and post-translational regulatory mechanisms, 20/38 of the transcript/protein matches were in opposite directions [78], while 18/38 had the same orientation. Consistent with the divergent premise, 100% of the transcript/protein pairs showed opposite directionality with a threshold of *p* ≤ 0.050.

### 3.11. Differential Expression of Glutamine Transporters and Glutamine Synthetase in SMA

**Figure 10:**
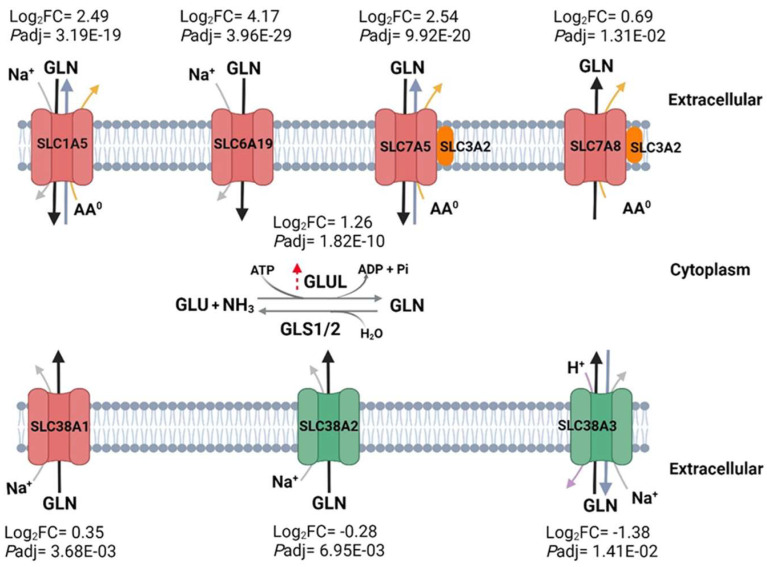
**Model showing significantly dysregulated glutamine transporters and glutamine synthetase.** The RNA-seq analyses from 66 children [non-SMA (n = 41) and SMA (n = 25)] containing *P*adj genes at <0.05 revealed dysregulation in seven human GLN transporters in children with SMA, five up-regulated (red) and two down-regulated (green). The functionality of both SLC7A5 and SLC7A8 relies on the formation of a complex with a chaperone, SLC3A2 (orange). The directionality of the arrows indicates the flux of GLN (black), Na^+^ (gray), neutral amino acids (orange), and H^+^ (purple). Conversion of GLU to GLN is catalyzed by GLUL (dotted red arrow indicating up-regulation), whereas conversion of GLN to GLU is catalyzed by GLS1/2 (not significantly altered). Log_2_FC (for Log_2_FoldChange) and *P*adj are shown for the significant DEGs. The complete gene list can be found in Table S4. Abbreviations and symbols: GLN: glutamine; AA^0^: neutral amino acids; Na^+^: sodium ion; H^+^: hydrogen ion; Glu: glutamate; GLUL: glutamate-ammonia ligase (i.e., glutamine synthetase); GLS1/2: glutaminase1/2. Created in BioRender.com (accessed on 20 August 2024). The unsupervised hierarchical clustering heatmap for glutamine transporters is shown in Figure S3.

Since GLN is a critical regulator of HSP responses and must be actively taken up by cells to counter the stress response induced by conditions such as SMA, GLN transporters were explored. Of the 18 characterized human GLN transporter genes, 7 were differentially regulated between the non-SMA and SMA groups (Table S4). SMA was characterized by up-regulation of five transporter genes: SLC6A19 (log_2_FC = 4.17), SLC7A5 (2.54), SLC1A5 (2.49), SLC7A8 (0.69), and SLC38A1 (0.35), and down-regulation of two transporter genes: SLC38A2 (−0.28) and SLC38A3 (−1.38) (Figure 10). Unsupervised hierarchical clustering of the seven transporters revealed two clusters, with the three most differentially up-regulated GLN transporters (~2.5 Log2FC) forming cluster 1 (i.e., SLC6A19, SLC7A5, and SLC1A5), while the remaining four transporters (two up-regulated and two down-regulated) formed cluster 2 (i.e., SLC7A8, SLC38A1, SLC38A2, and SLC38A3) (Figure S3). Children with SCA who developed SMA had intensified up- and down-regulation of the GLN transporters in clusters 1 and 2, respectively. To further explore cellular activity that can influence GLN levels, GLUL and GLS1/2 were investigated. Exploration of GLUL revealed that it was significantly up-regulated in children with SMA (*P*adj = 1.82 × 10^−10^, log_2_FC = 1.26, Figure 10 and Table S4). While GLS1 plays a central role in providing glutamate for the tricarboxylic acid (TCA) cycle, GLS1 and GLS2 are important in regulating cellular metabolism [55]. However, differing gene expression profiles were not witnessed for either GLS1 (*P*adj = 0.062, log_2_FC= −0.23) or GSL2 (*P*adj = 0.778, log_2_FC= 0.14, Table S4). These findings indicate that children with SMA exhibit up-regulation of GLN transporters to boost cellular GLN absorption and increased transcripts for a critical enzyme, GLUL, that increases GLN production. Key resources for all experiments are listed in Table S5.

## 4. Discussion

The etiology of SMA is multifaceted, primarily characterized by enhanced hemolysis and impaired erythropoiesis, which are, at least in part, related to dysregulation in innate immune responses [4]. For example, we have shown that children with SMA in the holoendemic *P. falciparum* transmission region in western Kenya, where the current investigations were performed, have imbalances in circulating cytokines and chemokines that are associated with inefficient erythropoiesis and severe anemia (e.g., IFN-γ, IL-6, IL-10, IL-12, IL-13, IL-21, IL-23, MIF, MIP-1α, MIP-1β, RANTES (CCL5), TGF-β1, and TNF-α) [5,79,80,81,82].

To extend these findings and move beyond the individual gene-level approach, we employed RNA-seq to capture the entire expressed transcriptome in whole blood. This approach revealed a substantial number of DEGs (3420 up-regulated and 3442 down-regulated) in children with SMA compared to non-SMA controls. The nearly equal distribution of up-regulated and down-regulated genes suggests a complex and multifaceted transcriptional response to SMA pathogenesis. Since it is well established that the immune response conditions malarial severity [4], deconvolution analysis was performed using CIBERSORTx to capture general immune profiles. This analysis revealed that SMA was characterized by a decreased antigenic response, reduced immune priming, and an enhanced polarization towards cellular proliferation and repair. While not directly comparable to our clinical groups, flow cytometric analyses of immune profiles in Ghanaian children with asymptomatic and symptomatic *P. falciparum* infections revealed significant changes in CD4 T cells, CD8 T cells, monocytes, and natural killer cells during acute symptomatic infections, aligning with results presented here [83]. Another study in Kenyan children utilized CIBERSORTx to deconvolute cell-type proportions from transcriptome data before, during, and after malaria episodes [84]. This investigation revealed significant changes in immune cells, including variations in CD8 T cells and memory CD4 T cells. These changes reflect immune dynamics associated with infection and recovery, generally consistent with the profiles identified in our clinical groups.

To explore the co-expressed genes that likely share biological and regulatory functions, WGCNA was performed on the entire expressed transcriptome dataset. This analysis revealed 21 distinct gene modules, with M15 and M19 showing statistically significant relationships with the clinical traits (i.e., non-SMA and SMA), suggesting their potential importance in the pathogenesis of SMA. Functional enrichment analysis revealed that M15 is strongly linked to immune regulation, including pathways involved in neutrophil degranulation, innate immune responses, and TLR cascades. This finding highlights a conglomeration of responses that regulate early (innate) immune activation. Similarly, M19 showed a significant connection to immune response mechanisms, particularly in protein interactions and cytokine signaling, indicating its importance in host defense processes. The WGCNA provided insight into the molecular underpinnings of SMA, with immune-regulated networks emerging as significant contributors to disease progression.

Since immune response networks emerged as the most enriched features that distinguished between the non-severe and severe clinical phenotypes, the pathogenesis of SMA was further explored by identifying the top 10-ranked canonical pathway maps for the immune response. This analysis revealed substantial dysregulation in the HSP60-HSP70-TLR2/4 signaling pathway as one of the top emergent features, supporting the findings from the WGCNA for M15. In particular, genes for multiple components of the HSP60-HSP70-TLR2/4 signaling pathway were down-regulated in children with SMA, such as HSP70 family members, HSP60, TLR2, TLR4, CD14, MD-2/LY96, MyD88, IRAK4, IRAK2, MEK6/MAP2K6, MAPK14, NF-kB1, NFKB2, IKKα/CHUK, I-κB/NFKBIA, JUNB, IL-6, NF-κB1/NFKB1, IL-1β, CD69, ICAM1, and MHC class I molecules. Conversely, other genes within the pathway were up-regulated, including IRAK1, TAB2, TAB3, ubiquitin B/UBB, UEV1A/UBE2V1, TAK1/MAP3K7, MEK2/MAP2K2, MEK3/MAP2K3, ERK2/MAPK1, JUND, CD86, CD80, and MHC class II molecules. The pattern of down-regulation of genes in the HSP60-HSP70-TLR2/4 signaling pathway (e.g., HSP70 family members, TLR2, TLR4, CD14, and NF-κB1) suggests that children with SMA may have reduced pattern recognition and inflammatory response. At the same time, up-regulation of other components (e.g., IRAK1, CD86, and MHC class II molecules) indicates an attempt to compensate through alternative immune activation and antigen presentation mechanisms [85,86]. Our results differ from those obtained in adults, both in malaria-naïve USA volunteers experimentally infected with *P. falciparum* and in naturally infected Cameroonian individuals with clinically apparent falciparum malaria, where GeneChip analysis (Affymetrix U133A) of PBMCs showed up-regulation of genes for HSP60, HSPA1A, HSPA1B, HSPA4, HSPA5, HSPA9B, TLR2, TLR4, CD14, MYD88, IRAK1, IKKα, NF-κB1, IL-1β, and ICAM1 [87]. The differences between gene expression patterns in children with SMA and adults with malaria may be due to variations in immune system maturity, disease severity, experimental conditions, pathogen strain differences, and methodological approaches.

Our previous study showed that leukocytic HSP70 leukocytic transcripts of HSP70 (HSPA1A and HSPA1B) were significantly reduced in children with SMA and positively correlated with the reticulocyte production index and Hb concentrations [12]. Here, we expanded those results by linking suppression of HSP70 with TLR2 and TLR4, HSP60, and associated signaling cascades in malaria-infected children with severe anemia. HSP70 plays an essential role in erythropoiesis by protecting an erythroid transcription factor, GATA-1, from caspase-3-mediated proteolysis at later stages of erythroblast maturation [88]. Moreover, HSP70 may play a role in the severe anemia witnessed in individuals with β-Thalassemia (β-TM) since HSP70 directly interacts with free αglobin chains in human β-TM erythroblasts, resulting in HSP70 sequestration in the cytoplasm, a process that promotes GATA-1 degradation [89]. HSP70 and HSP60 are also essential for appropriate erythropoietic responses by aiding in protein folding, protecting erythroid precursors from stress-induced apoptosis during hypoxia and iron deficiency, supporting mitochondrial function, and regulating the differentiation and proliferation of the erythroid lineage [90,91,92]. Thus, our previous and current findings offer novel insight into how dysregulation in the HSP60-HSP70-TLR2/4 signaling pathway may contribute to inefficient erythropoiesis and the profound, life-threatening anemia witnessed in children with SMA.

Since the inheritance of sickle cell trait (HbAS) has protective effects against the development of severe malaria, children with SMA have a higher proportion of HbSS carriage, particularly in holoendemic regions [77,85,93,94,95]. To capture this natural demographic, children with all sickle cell genotypes were included in the primary transcriptomic analyses, followed by secondary analyses with the removal of HbSS carriers from the non-SMA and SMA groups. Significant immune dysregulation in the HSP60-HSP70-TLR2/4 signaling pathway was witnessed in the SMA group with or without SCA, indicating that this pathway is a characteristic feature of SMA, independent of HbSS carriage.

The RNA-seq data for DEGs in the HSP60-HSP70-TLR2/4 signaling pathway was validated using global gene expression profiling (>19,185 transcripts, Illumina^®^ HumanHT-12 v4 beadchip) on whole blood samples from a separate cohort of children with non-SMA (n = 38, average Hb = 9.3 g/dL) and SMA (n = 12, average Hb = 5.3 g/dL). The global gene expression profiling confirmed the DEGs in the HSP60-HSP70-TLR2/4 signaling pathway with a high significance level, underscoring the importance of the immune response pathway in the pathogenesis of SMA. Although a comparable dataset was not located for external validation, a comprehensive transcriptomic analysis was recently conducted using PBMCs from Kenyan children, including 21 asymptomatic–febrile pairs and 22 uninfected–febrile pairs [96]. This investigation revealed that febrile infections were characterized by the up-regulation of immune pathways related to immune effector functions, production of inflammatory cytokines, and humoral responses. Despite comparable panels of genes being captured, a direct comparison of the expression patterns for the HSP60-HSP70-TLR2/4 signaling pathway and CIBERSORTx results was challenging since the febrile children were not presented according to disease severity measures.

To evaluate the relationship between DEGs in the HSP60-HSP70-TLR2/4 signaling pathway and protein levels, transcriptomic data from whole blood was compared with plasma protein abundance using the 7k SomaScan platform for 35 children (non-SMA n = 19, SMA n = 16) who had both RNA-seq and proteomic data. Integration of the RNA-seq and proteome data for the HSP60-HSP70-TLR2/4 signaling pathway revealed highly significant regulatory changes in protein levels in children with SMA. These data revealed enrichment for the expected differences in directional patterns between the transcript/protein pairs in samples measured at the same sample collection time. To fully capture the molecular relationship between transcript/protein pairs in the HSP60-HSP70-TLR2/4 signaling pathway, serially collected samples must be measured in future studies to account for post-transcriptional and post-translational modifications.

We have previously shown that reduced circulating GLN levels are a significant predictor of SMA and that GLN treatment can overcome hemozoin-induced suppression of HSP70 transcripts and protein in human PBMCs from malaria-naïve donors [12]. However, the etiology of reduced GLN in children with SMA remains undetermined. Multiple functions have been documented for GLN in mammalian cells, including leukocytes through the following: (1) cellular signaling by activating heat shock factor 1 (HSF1) and suppressing NF-κB activity; (2) a crucial energy source, through its involvement in the TCA cycle; and (3) synthesis of biomolecules, including nucleotides and non-essential amino acids (NEAA), such as glutamate, asparagine, aspartate, and alanine, as well as proteins and uridine diphosphate N-acetylglucosamine (UDP-GlcNAc), which are important for protein post-translational modifications [40,97,98,99,100]. Furthermore, GLN has been shown to facilitate erythropoiesis by providing succinyl-CoA for heme synthesis [101]. Potential reasons for reduced GLN in children with SMA, inferred from findings in other non-malarial studies, include increased metabolic demands, where GLN is rapidly consumed via the TCA cycle, and overactivation of the immune response, which significantly consumes GLN for the proliferation and function of immune cells [40]. In addition, children in holoendemic falciparum regions often suffer from nutritional deficiencies in which inadequate protein intake can lead to low levels of amino acids, including GLN [40]. Children with severe malaria may also have impaired liver function, reducing the synthesis and availability of GLN since the liver is a key site for its production [40]. Children with SMA also have enhanced oxidative stress that necessitates the utilization of GLN for the synthesis of glutathione (GSH) and NADPH, which are essential for maintaining the cellular redox balance [40]. Lastly, children with SMA often suffer from acute kidney injury, which impairs glutamine (GLN) metabolism, leading to increased oxidative stress and apoptosis in tubular epithelial cells [102,103].

The uptake of GLN into cells, including lymphocytes and macrophages, to facilitate its increased demand requires transporters on the cell surface [104]. The primary difference in GLN transporters lies in their specific functions, substrate specificities, and tissue distributions (see Figure 6) [51,105,106]. The significant up-regulation of SLC1A19, SLC7A5, SLC1A5, and SLC7A8 may be related to an increased metabolic demand in the high-stress state of SMA in which enhanced energy metabolism and synthesis of nucleotides are required [40,97,98,99,100]. The increase in these specific transporters in SMA is also likely related to an increased need for GLN to support the proliferation and activity of immune cells and an enhanced requirement for cellular repair, antioxidant defense, and cellular survival mechanisms [40,51,105,107]. SLC1A5 is essential for Th1 and Th17 cell production and inflammatory T-cell responses [108]. Consistent with our findings, a previous study in Gabonese children revealed that SLC6A19 transcripts were highly up-regulated in SMA relative to those with uncomplicated malaria [109]. In addition to the up-regulation of specific GLN transporters, two transporters were significantly down-regulated: SLC38A2 and SLC38A3. Given decreased GLN levels in children with SMA, down-regulation of these GLN transporters is expected to compromise GLN uptake into cells, impairing immune cell function, antioxidant defense, energy metabolism, and nitrogen balance, a pattern of responsiveness that could weaken the ability to respond to and recover from the malarial infection [40,51,105]. The formation of two distinct clusters in the unsupervised hierarchical clustering suggests that the three most differentially expressed up-regulated genes (cluster 1) may have coordinated regulation driven by a common mechanism. The mixed regulation in cluster 2 indicates a more complex and nuanced response, potentially reflecting different functional roles/regulatory mechanisms in the disease process. In addition to GLN uptake through transporters, the combined actions of GLUL and GLS/GLS2 also control intracellular GLN levels [110,111] While GLUL was significantly up-regulated in SMA, GLS1/2 did not differ between the groups. Up-regulation of *GLUL* transcription likely indicates a compensatory mechanism to enhance leukocytic GLN levels in SMA.

Since this study focuses on Kenyan children (aged 1–59 mos.) with SMA, there could be limited generalizability to other populations in geographic regions who suffer from SMA or different forms of severe malaria (e.g., cerebral malaria). We could not locate publicly available sources containing data for transcripts and proteins in the HSP60-HSP70-TLR2/4 signaling pathway to validate our findings in other populations of children. The cross-sectional design hinders causal inferences, and a single-time-point analysis may not capture dynamic changes in gene expression and/or the molecular relationship between transcript/protein pairs. Additional functional studies are required to understand the roles of dysregulated genes and pathways studied here. Future research should address these gaps.

## 5. Conclusions

Exploring the entire expressed transcriptome revealed that key genes in the HSP60-HSP70-TLR2/4 signaling pathway, as well as genes for GLN transporters and GLN metabolizing enzymes, are significantly altered in SMA. This includes down-regulation of key protective and immune response genes, such as those coding for HSPs, TLRs, and certain MHC class I components, alongside the mixed regulation of MHC class II, TLR signaling components, GLN transporters, and GLN metabolizing enzymes. Since the inheritance of sickle cell trait (HbAS) has protective effects against the development of severe malaria, children with SMA have a higher proportion of HbSS carriage, particularly in holoendemic regions. To capture this natural demographic, children with all sickle cell genotypes were included in the primary transcriptomic analyses, followed by secondary analyses with the removal of HbSS carriers from the non-SMA and SMA groups. Significant immune dysregulation in the HSP60-HSP70-TLR2/4 signaling pathway was identified in the SMA group with or without SCA, indicating that this pathway is a characteristic feature of SMA, independent of HbSS carriage. These findings highlight the critical role of these signaling pathways in modulating inflammation and immune responses in SMA, suggesting potential targets for therapeutic intervention and demonstrating that immune dysregulation in the HSP60-HSP70-TLR2/4 signaling pathway is central to SMA pathogenesis, regardless of sickle cell status.

## Figures and Tables

**Table 1:** Demographic, clinical, and laboratory characteristics of the study participants.

Characteristics	Non-SMA (Hb ≥ 6.0 g/dL)	SMA (Hb < 6.0 g/dL)	*p-*Value
No. of participants (n = 66)	41	25	
Sex, n (%)			
Male	20 (48.8)	13 (52.0)	0.800 ^a^
Female	21 (51.2)	12 (48.0)	
Age, months	24.0 (22.0)	25.0 (28.5)	0.797 ^b^
0–12.9	7 (17.1)	5 (20.0)	0.461 ^a^
13–24.9	14 (34.1)	7 (28.0)	
25–35.9	9 (22.0)	5 (20.0)	
36–48.9	11 (26.8)	6 (24.0)	
≥49	0 (0.0)	2 (8.0)	
Blood glucose, mmol/L	5.0 (2.3)	5.0 (1.7)	0.967 ^b^
Temporal temperature, °C	38.0 (1.2)	37.7 (0.8)	0.051 ^b^
**Hematological Parameters**			
Hemoglobin, g/dL	9.9 (1.4)	4.6 (1.2)	NA
Hematocrit, %	29.8 (5.9)	14.4 (2.9)	**1.242 × 10^−11 b^**
Red blood cells, ×10^6^/µL	4.3 (1.0)	1.9 (0.9)	**1.790 × 10^−11 b^**
Red blood cell distribution width, %	18.7 (3.4)	22.3 (8.9)	**4.050 × 10^−4 b^**
Mean corpuscular volume, fL	69.5 (9.2)	78.6 (29.9)	**0.002 ^b^**
Mean corpuscular hemoglobin, pg	22.9 (4.8)	26.7 (9.4)	**0.022 ^b^**
Platelets, ×10^3^/µL	124.4 (85.7)	134.0 (139.7)	0.615 ^b^
Platelet distribution width, %	16.5 (1.3)	17.3 (0.9)	0.370 ^b^
Mean platelet volume, fL	8.5 (1.6)	8.9 (1.9)	0.124 ^b^
WBCs, ×10^3^/µL	11.3 (6.9)	19.8 (11.5)	**1.393 × 10^−4 b^**
Lymphocytes, ×10^3^/µL	3.7 (1.9)	10.0 (9.2)	**5.509 × 10^−6 b^**
Monocytes, ×10^3^/µL	1.2 (1.3)	1.7 (1.4)	**0.022 ^b^**
Neutrophils, ×10^3^/µL	5.3 (4.2)	6.0 (6.9)	0.438 ^b^
Granulocytes, ×10^3^/µL	6.7 (3.0)	9.1 (5.8)	0.373 ^b^
**Parasitological Indices**			
Parasite density, MPS/µL	57,915 (81,568)	14,191 (68,728)	0.155 ^b^
Low (1–5000)	6 (14.6)	8 (32.0)	0.134 ^a^
Moderate (5001–50,000)	13 (31.7)	10 (40.0)	
High (50,001–100,000)	14 (34.1)	3 (12.0)	
Hyper (>100,001)	8 (19.5)	4 (16.0)	
**Genetic Variants**			
Sickle-cell genotypes, n (%)			
HbAA	35 (85.3)	16 (64.0)	
HbAS	4 (9.8)	2 (8.0)	**0.029 ^a^**
HbSS	2 (4.9)	7 (28.0)	

Unless otherwise noted, data are presented as the median (interquartile range; IQR). Children (n = 66) presenting with malaria were recruited at SCRH. Based on hemoglobin (Hb) levels, children were categorized into either non-severe malarial anemia (non-SMA; Hb ≥ 6.0 g/dL, n = 41) or severe malarial anemia (SMA; Hb < 6.0 g/dL, n = 25). ^a^ Fisher’s exact test with exact *p*-values for homogeneity and ^b^ Mann–Whitney U test were used to compare the non-SMA and SMA groups. Statistical significance was set at *p* ≤ 0.050; significant *p-*values are indicated in bold. Abbreviations: MPS: malaria parasites; HbAA: hemoglobin AA; HbAS: hemoglobin AS; HbSS: hemoglobin SS. Blue color indicates characteristic categories.

## Data Availability

The RNA sequencing data and associated metadata that support the findings of this study have been deposited in the National Library of Medicine (NLM) Gene Expression Omnibus (GEO) and are publicly accessible under the accession number GSE255403. The data include raw sequencing reads, processed gene expression matrices, and detailed experimental annotations. These resources are available to the research community to ensure transparency and reproducibility of our results and to facilitate further investigations into the genetic underpinnings of severe malarial anemia. Researchers can access the dataset directly through the GEO repository at https://www.ncbi.nlm.nih.gov/geo/query/acc.cgi?acc=GSE255403, accessed on 9 September 2024. Additional information regarding the proteomics measures can be found in Anyona S.B., Cheng Q., Wasena S.A., Osata S.W., Guo Y., Raballah E., Hurwitz I., Onyango C.O., Ouma C., Seidenberg P.D., et al. (2024). Entire expressed peripheral blood transcriptome in pediatric severe malarial anemia. Nat. Commun. 15, 5037. doi:10.1038/s41467-024-48259-4. Any additional information required to reproduce the findings or queries regarding the data should be directed to the corresponding author.

## Supplementary Materials

The following supporting information can be downloaded at the following site: https://www.mdpi.com/article/10.3390/pathogens13100867/s1, Table S1. Differentially expressed transcripts in the HSP60-HSP70-TLR2/4 signaling pathway in children with severe malarial anemia; Table S2. Top process networks of DEGs in clusters 1 and 2 of the heatmap; Table S3. Demographic, clinical, and laboratory characteristics of the study participants in the validation cohort; Table S4. Differential expression of glutamine transporters and glutamine synthetase transcripts in children with severe malarial anemia; Table S5. Key resources table; Figure S1. HSP60-HSP70-TLR2/4 signaling pathway in children without sickle cell anemia; Figure S2. Overall study design, data collection, and sampling strategy for transcriptomic validation; Figure S3. Unsupervised hierarchical clustering heatmap for glutamine transporters.
