# Supplementary material for: Transcriptomic and Proteomic Insights into Host Immune Responses in Pediatric Severe Malarial Anemia: Dysregulation in HSP60-70-TLR2/4 Signaling and Altered Glutamine Metabolism
Source: Pathogens. 2024 Oct 3;13(10):867. doi: 10.3390/pathogens13100867 (PMC11510049; doi:10.3390/pathogens13100867)
Supplement: Supplementary file 1 [file pathogens-13-00867-s001.zip › pathogens-3170396-supplementary.pdf]

SUPPLEMENTARY MATERIALS

Transcriptomic and Proteomic Insights into Host Immune Responses in Pediatric Severe Malarial Anemia: Dysregulation in HSP60-70-TLR2/4 Signaling and Altered Glutamine Metabolism

Clinton O. Onyango[1,2], Samuel B. Anyona[3,4], Ivy Hurwitz[2], Evans Raballah[3,5], Sharely A. Wasena[1,2], Shamim W. Osata[1,2], Philip Seidenberg[2,6], Benjamin H. McMahon[2,7], Christophe G. Lambert[2,8], Kristan A. Schneider[2,8], Collins Ouma[1,2], Qiuying Cheng[2,3,*], and Douglas J. Perkins[2,3,*]

Table S1. Differentially expressed transcripts in the HSP60-HSP70-TLR2/4 signaling pathway in children with severe malarial anemia.

Table S2. Top process networks of DEGs in clusters 1 and 2 of the heatmap.

Figure S1. HSP60-HSP70-TLR2/4 signaling pathway in children without sickle cell anemia.

Figure S2. Overall study design, data collection, and sampling strategy for transcriptomic validation.

Table S3: Demographic, clinical, and laboratory characteristics of the study participants in the validation cohort.

Table S4. Differential expression of glutamine transporters and glutamine synthetase transcripts in children with severe malarial anemia.

Figure S3. Unsupervised hierarchical clustering heatmap for glutamine transporters.

Table S5. Key resources table.

Table S1. Differentially expressed transcripts in the HSP60-HSP70-TLR2/4 signaling pathway in children with severe malarial anemia.

Gene	Log_2foldchange	*P*adj
HSP60		
HSPD1	-0.81	**1.37E-05**
HSP70		
HSPA1A	-1.31	**1.99E-04**
HSPA1B	-0.99	**9.23E-03**
HSPA4	-0.33	**2.27E-02**
HSPA4L	-0.68	**3.49E-02**
HSPA5	-0.51	**1.33E-03**
HSPA6	-0.81	**1.88E-03**
TLR2	-0.53	**1.94E-02**
TLR4	-0.83	**1.29E-04**
CD14	-0.60	**1.07E-02**
MD-2 (LY96)	-0.89	**1.13E-05**
TIRAP(Mal)	0.06	0.670
MYD88	-0.32	**4.60E-02**
IRAK1	0.23	**2.31E-02**
IRAK2	-0.81	**1.08E-04**
IRAK4	-0.36	**1.66E-05**
TRAF6	-0.05	0.581
E2N (UBC13, UBE2N)	-0.10	0.257
UEV1A (UBE2V1)	0.45	**7.99E-03**
TAB1	0.20	0.122
TAB2	0.57	**1.51E-05**
TAB3	1.82	**3.70E-17**
Ubiquitin B (UBB)	0.94	**1.29E-03**
UBE1 (UBA1)	-0.16	0.156
TAK1(MAP3K7)	0.18	**4.97E-02**
MEK1 (MAP2K1)	-0.21	0.057
MEK2 (MAP2K2)	0.55	**1.36E-05**
MEK3 (MAP2K3)	1.44	**7.36E-09**
MEK4 (MAP2K4)	0.11	0.360
MEK6 (MAP2K6)	-0.68	**3.08E-03**
TPL2 (MAP3K8)	-0.13	0.322
NF-kB1 (p105) [NFKB1]	-0.40	**6.97E-04**
IKK-alpha (CHUK)	-0.49	**1.24E-06**
IKK-beta (IKBKB)	-0.08	0.230
IKK-gamma (IKBKG)	0.18	0.211
JNK (MAPK8)	-0.01	0.980
p38 MAPK (MAPK14)	-0.78	**7.52E-05**
ERK1 (MAPK3)	-0.16	0.426
ERK2 (MAPK1)	0.60	**2.30E-06**
AP-1		
JUNB	-0.57	**6.93E-03**
JUND	1.95	**2.31E-19**
c-Jun (JUN)	-0.12	0.677
c-Fos (FOS)	-0.19	0.537
NFKB2	-0.66	**6.40E-04**
RELB	-0.32	**4.98E-02**
I-κB (NFKBIA)	-0.91	**1.08E-04**
IL-1β	-1.51	**4.54E-11**
IL-6	-0.60	**3.56E-02**
IL-8 (CXCL8)	-0.55	0.262
IL-10	-0.23	0.513
IL-12 alpha chain (IL12A)	-0.15	0.535
IL-12 beta chain (IL12B)	-0.26	0.621
TNF-alpha (TNF)	-0.35	0.100

iNOS (NOS2)	-0.48	0.485
CD40	-0.08	0.702
CD69	-0.29	**3.78E-02**
CD80	1.54	**1.88E-13**
CD83	-0.32	0.084
CD86	0.54	**6.41E-03**
ICAM1	-1.04	**6.68E-07**
MHC Class I		
HLA-B	-0.33	**2.34E-02**
HLA-C	-0.54	**7.71E-04**
HLA-E	-0.39	**2.43E-03**
B2M	-0.37	**6.93E-03**
MHC Class II		
HLA-DMA	0.38	**2.94E-03**
HLA-DOA	0.70	**1.11E-04**
HLA-DOB	0.42	**2.06E-02**
HLA-DPB1	0.56	**2.00E-03**
HLA-DPB2	0.75	**2.03E-02**

Student's *t*-test was used to compare the mean gene expression level between non-SMA (Hb≥6.0 g/dL, n=41) and SMA (Hb<6.0 g/dL, n=25). The Log_2foldchange was defined as the Log base 2 value of the ratio of the mean gene expression level in SMA to that of non-SMA. Bold indicates statistical significance at *P*adj≤0.050.

Table S2. Top process networks of DEGs in clusters 1 and 2 of the heatmap.

Rank		Networks	Total	In Data	P*adj*	Network Objects from Active Data
Cluster 1 (down-regulated)	1	Inflammation-Amphoterin signaling	118	21	1.192E-25	IL-1 beta, TLR2, NF-kB2 (p100), IRAK2, I-kB, NF-kB, NF-kB1 (p105), NFKBIA, IL-6, IRAK1/2, IKK-alpha, TLR4, NF-kB2 (p52), ICAM1, AP-1, p38alpha (MAPK14), p38 MAPK, NF-kB1 (p50), NF-kB p50/p50, MyD88, NF-kB p52/RelB
	2	Inflammation-Innate inflammatory response	181	23	3.163E-25	IL-1 beta, TLR2, sCD14, NF-kB2 (p100), IRAK2, I-kB, NF-kB, IRAK4, NFKBIA, IL-6, IRAK1/2, IKK-alpha, TLR4, NF-kB2 (p52), p38alpha (MAPK14), p38 MAPK, NF-kB1 (p50), NF-kB p50/p50, MyD88, NF-kB p52/RelB, CD14, MD-2, HSPA1A
	3	Inflammation-IL-10 anti-inflammatory response	87	17	2.124E-21	IL-1 beta, NF-kB2 (p100), I-kB, NF-kB, NFKBIA, IL-6, IRAK1/2, IKK-alpha, TLR4, NF-kB2 (p52), p38alpha (MAPK14), MEK6(MAP2K6), p38 MAPK, NF-kB1 (p50), NF-kB p50/p50, MyD88, NF-kB p52/RelB
	4	Immune Response-Antigen presentation	193	19	2.308E-18	NF-kB2 (p100), HLA-E, I-kB, Beta-2-microglobulin, NF-kB, NF-kB1 (p105), NFKBIA, IKK-alpha, GRP78, NF-kB2 (p52), HLAB, ICAM1, HSP70, NF-kB1 (p50), RelB (NF-kB subunit), NF-kB p50/p50, NF-kB p52/RelB, HLAC, MHC class I
	5	Immune Response-Phagosome in antigen presentation	241	20	4.655E-18	NF-kB2 (p100), I-kB, Beta-2-microglobulin, NF-kB, NF-kB1 (p105), NFKBIA, IKK-alpha, GRP78, TLR4, NF-kB2 (p52), HLAB, p38alpha (MAPK14), p38 MAPK, HSP70, NF-kB1 (p50), NF-kB p50/p50, NF-kB p52/RelB, HLAC, MHC class I, CD14
	6	Inflammation-MIF signaling	141	17	6.385E-18	IL-1 beta, NF-kB2 (p100), IRAK2, I-kB, NF-kB, NFKBIA, IL-6, IRAK1/2, IKK-alpha, TLR4, NF-kB2 (p52), ICAM1, NF-kB1 (p50), NF-kB p50/p50, MyD88, NF-kB p52/RelB, CD14
	7	Inflammation-Protein C signaling	108	15	1.133E-16	IL-1 beta, NF-kB2 (p100), IRAK2, I-kB, NF-kB, NFKBIA, IL-6, IRAK1/2, IKK-alpha, TLR4, NF-kB2 (p52), NF-kB1 (p50), NF-kB p50/p50, MyD88, NF-kB p52/RelB
	8	Inflammation-TREM1 signaling	145	16	2.872E-16	IL-1 beta, TLR2, IRAK2, I-kB, NF-kB, NFKBIA, IL-6, IRAK1/2, IKK-alpha, TLR4, ICAM1, p38alpha (MAPK14), p38 MAPK, MyD88, CD14, MD-2
	9	Cell Cycle-G1-S Interleukin regulation	128	15	1.240E-15	IL-1 beta, NF-kB2 (p100), I-kB, NF-kB, NFKBIA, IRAK1/2, IKK-alpha, NF-kB2 (p52), AP-1, p38alpha (MAPK14), NF-kB1 (p50), RelB (NF-kB subunit), NF-kB p50/p50, NF-kB p52/RelB, JunB
	10	Immune response-TCR signaling	174	16	4.464E-15	NF-kB2 (p100), I-kB, NF-kB, NF-kB1 (p105), NFKBIA, IKK-alpha, NF-kB2 (p52), ICAM1, AP-1, p38alpha (MAPK14), MEK6(MAP2K6), p38 MAPK, NF-kB1 (p50), NF-kB p50/p50, NF-kB p52/RelB, MHC class I

**Cluster 2 (up-regulated)**	1	Immune Response-T helper cell differentiation	140	10	1.061E-10	CD86, MEK3(MAP2K3), ERK1/2, MEK2(MAP2K2), TAB2, TAK1(MAP3K7), CD80, IRAK1/2, IRAK1, MHC class II
	2	Immune Response-TCR signaling	174	10	4.764E-10	AP-1, CD86, MEK3(MAP2K3), ERK1/2, MEK2(MAP2K2), ERK2 (MAPK1), CD80, MEK1/2, Ubiquitin, MHC class II
	3	Inflammation-IL-4 signaling	115	8	1.294E-08	CD86, ERK1/2, MEK2(MAP2K2), HLA-DPB1, ERK2 (MAPK1), CD80, MEK1/2, MHC class II
	4	Inflammation-Amphoterin signaling	118	8	1.294E-08	AP-1, ERK1/2, MEK2(MAP2K2), ERK2 (MAPK1), TAK1(MAP3K7), IRAK1/2, IRAK1, MEK1/2
	5	Inflammation-TREM1 signaling	145	8	5.407E-08	CD86, ERK1/2, MEK2(MAP2K2), ERK2 (MAPK1), TAK1(MAP3K7), IRAK1/2, IRAK1, MEK1/2
	6	Inflammation-Inflammasome	120	7	3.772E-07	AP-1, MEK3(MAP2K3), ERK1/2, TAB2, ERK2 (MAPK1), TAK1(MAP3K7), IRAK1
	7	Signal Transduction-TGF-beta, GDF and Activin signaling	154	7	1.826E-06	AP-1, MEK3(MAP2K3), ERK1/2, TAB2, ERK2 (MAPK1), TAK1(MAP3K7), JunD
	8	Signal Transduction-CREM pathway	98	6	2.607E-06	AP-1, ERK1/2, MEK2(MAP2K2), ERK2 (MAPK1), MEK1/2, Ubiquitin
	9	Immune Response-Antigen presentation	193	7	6.656E-06	CD86, MHC class II beta chain, HLA-DPB1, CD80, HLA-DOA, HLA-DMA, MHC class II
	10	Translation-Regulation of initiation	127	6	9.282E-06	MEK3(MAP2K3), ERK1/2, MEK2(MAP2K2), ERK2 (MAPK1), TAK1(MAP3K7), MEK1/2

Top 10-ranked process networks generated in MetaCore™ for cluster 1 (down-regulated) and cluster 2 (up-regulated) DEGs. Process networks are represented, along with the total number of genes in the network (total), DEGs in the dataset for SMA (in data), rank of the network (P*adj*), and actual genes in the network (network objects from active data).

Figure S1

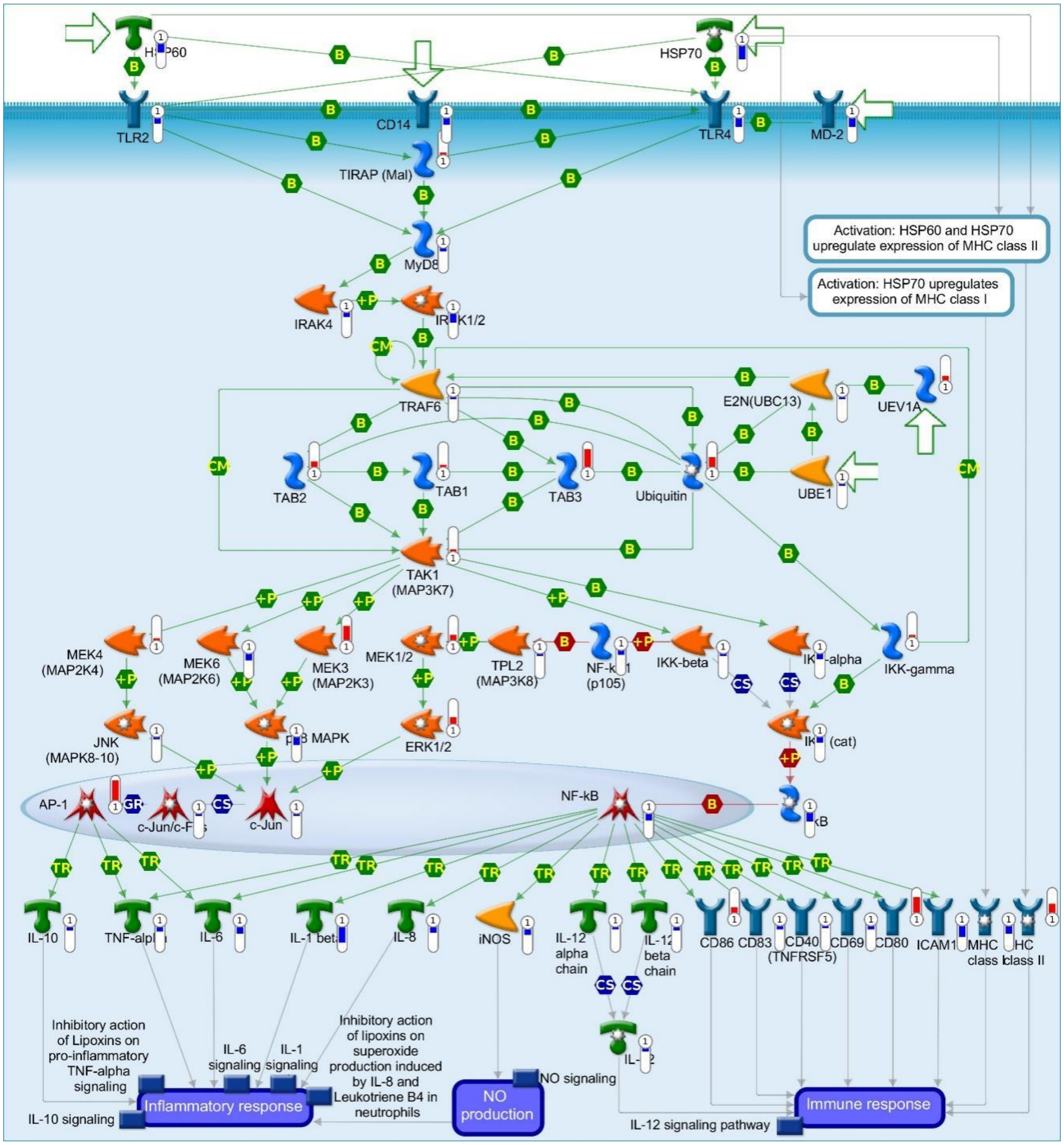

Figure S1. HSP60-HSP70-TLR2/4 signaling pathway in children without sickle cell anemia. Children with sickle cell anemia (HbSS) were excluded from the RNA-seq dataset of 66 children, which yielded a total of 39 non-SMA and 18 SMA. The canonical pathway generated with MetaCore™ mapped to 54/54 nodes (*P*adj=9.041E-13). The details of symbols used in these figures are available at: https://portal.genego.com/legends/MetaCoreQuickReferenceGuide.pdf.

Figure S2

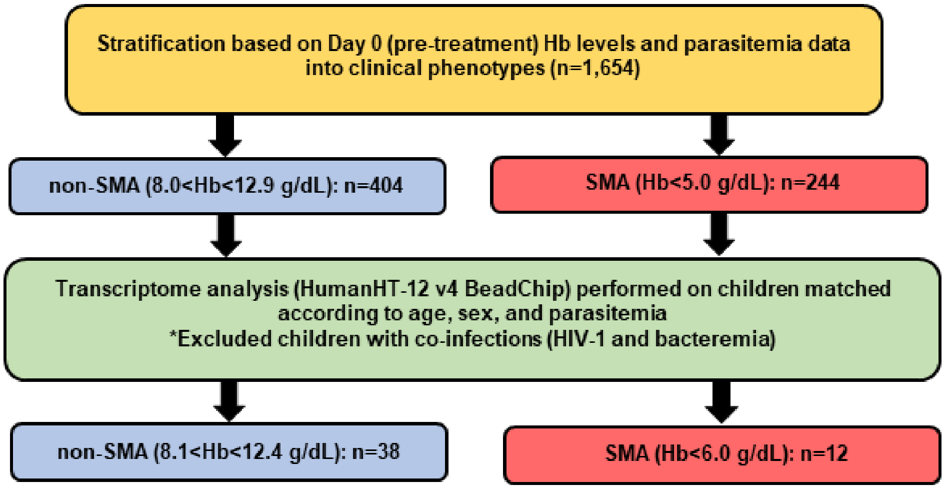

Figure S2. Overall study design, data collection, and sampling strategy for transcriptomic validation. The longitudinal study at Siaya County Referral Hospital (SCRH) recruited children (n=1,654, primarily <12 months) presenting at the hospital with acute febrile illness between 4/2004 to 9/2015. The longitudinal follow-up was 36 mos. Upon enrollment (Day 0), data were obtained for demographics, geospatial information, clinical status, and laboratory results. Prior to treatment interventions (i.e., antimalarials and other medications), venipuncture blood samples (3-4 mL) were obtained. Samples (Day 0, pretreatment) used for the Illumina® *"iScanSQ"* platform were selected to represent "polarized extremes" of clinical malaria phenotypes: non-SMA (Hb, 8.1-12.4 g/dL; n=38, avg. Hb=9.3 g/dL)] and SMA (Hb<6.0g/dL; n=12, avg. Hb=4.1 g/dL). Children were matched by age, sex, and parasitemia, excluding those with co-infections (i.e., HIV-1 and bacteremia).

Table S3: Demographic, clinical, and laboratory characteristics of the study participants in the validation cohort.

Characteristics	Non-SMA (Hb≥6.0g/dL)	SMA (Hb<6.0g/dL)	*P*-value
No. of participants (n=50)	38	12	
Sex, n (%)			
Male	20 (52.6)	5 (41.7)	0.742[a]
Female	18 (47.4)	7 (58.3)	
Age, months	8.5 (7.6)	11.7 (8.8)	0.296[b]
0 - 12.9	28 (73.7)	8 (66.7)	
13 - 24.9	10 (26.3)	4 (33.3)	
25 - 35.9	-	-	0.718[a]
36 - 48.9	-	-	
>49	-	-	
Blood glucose, mmol/L	6.6 (1.7)	6.3 (1.8)	0.474[b]
Temporal temperature, °C	38.0 (0.2)	38.0 (0.4)	0.617[b]
**Hematological Parameters**			
Hemoglobin, g/dL	9.3 (1.4)	5.3 (0.7)	NA
Hematocrit, %	30.3 (3.8)	18.9 (3.7)	**4.305E-07[b]**
Red blood cells, × 10^6/μL	4.5 (0.9)	2.7 (0.8)	**9.803E-07[b]**
Red blood cell distribution width, %	18.6 (5.0)	23.1 (5.6)	**0.008[b]**
Mean corpuscular volume, fL	70.1 (11.8)	69.3 (14.3)	0.641[b]
Mean corpuscular hemoglobin, pg	21.4 (4.7)	19.6 (4.6)	**0.022[b]**
Platelets, ×10^3/μL	176.0 (184.0)	158.0 (74.0)	0.064[b]
Platelet distribution width, %	17.3 (1.5)	17.8 (2.1)	0.370[b]
Mean platelet volume, fL	7.7 (1.5)	9.0 (4.1)	0.228[b]
WBCs, ×10^3/μL	12.5 (6.6)	15.8 (6.6)	**0.049[b]**
Lymphocytes, ×10^3/μL	5.5 (3.9)	7.8 (3.0)	**0.008[b]**
Monocytes, ×10^3/μL	0.9 (0.8)	1.7 (0.9)	**0.002[b]**
Neutrophils, ×10^3/μL	-	-	-
Granulocytes, ×10^3/μL	5.3 (4.4)	5.7 (4.0)	0.991[b]
**Parasitological Indices**			
Parasite density, MPS/μL	39,762 (145,905)	55,174 (171,007)	0.413[b]
Low (1 - 5,000)	8 (21.0)	1 (8.3)	
Moderate (5001 - 50,000)	13 (34.2)	5 (41.7)	0.703[a]
High (50,001 - 100,000)	5 (13.2)	1 (8.3)	
Hyper (>100,001)	12 (31.6)	5 (41.7)	
**Genetic Variants**			
Sickle-cell genotypes, n (%)			
HbAA	28 (75.7)	12 (100.0)	
HbAS	9 (24.3)	0 (0.0)	0.090[a]
HbSS	-	-	

Data are presented as the median (interquartile range; IQR) unless otherwise noted. Children (n=50) presenting with malaria were recruited at SCRH. Based on hemoglobin (Hb) levels, children were categorized into either non-severe malarial anemia (non-SMA; Hb≥6.0 g/dL, n=38) or severe malarial anemia (SMA; Hb<6.0 g/dL, n=12). [a]Fisher's exact test with exact *P*-values for homogeneity and [b]Mann-Whitney-U test were used to compare the non-SMA and SMA groups. Statistical significance was set at *P*≤0.050, and significant *P*-values are indicated in bold. Abbreviations: MPS - malaria parasites.

Table S4. Differential expression of glutamine transporters and glutamine synthetase transcripts in children with severe malarial anemia.

	**Gene**	**Log$_2$foldchange**	***P*adj**
**GLN Transporters**	SLC6A19	4.17	**3.96E-29**
	SLC7A5	2.54	**9.92E-20**
	SLC1A5	2.49	**3.19E-19**
	SLC7A8	0.69	**1.31E-02**
	SLC38A1	0.35	**3.68E-03**
	SLC38A2	-0.28	**6.95E-03**
	SLC38A3	-1.38	**1.41E-02**
	SLC6A14	-0.86	0.197
	SLC7A6	0.21	0.068
	SLC7A7	0.11	0.665
	SLC7A9	-0.44	0.320
	SLC38A4	-0.25	0.656
	SLC38A5	-0.14	0.729
	SLC38A6	0.19	0.391
	SLC38A7	0.06	0.699
	SLC38A8	-0.79	0.202
	SLC38A9	-0.15	0.175
	SLC38A10	-0.09	0.513
**GLN Enz**	GLUL	1.26	**1.82E-10**
	GLS (GLS1)	-0.23	0.062
	GLS2	0.14	0.778

Student's *t*-test was used to compare the mean gene expression level between non-SMA (Hb≥6.0 g/dL, n=41) and SMA (Hb<6.0 g/dL, n=25). The Log$_2$foldchange was defined as the ratio of mean gene expression level in SMA to that in non-SMA. Bold indicates statistical significance at *P*adj≤0.050.

Figure S3

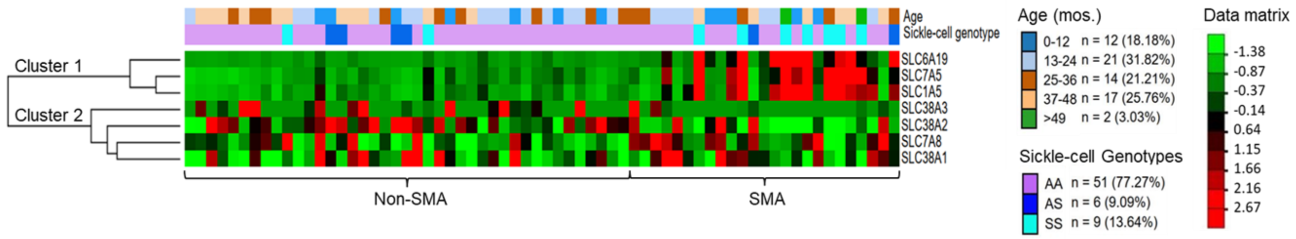

Figure S3. Unsupervised hierarchical clustering heatmap for glutamine transporters. Expression values for each of the 7 DEGs selected based on a *P*adj≤0.050 (rows) were normalized across all samples (columns). The dendrogram of hierarchical clustering of genes was based on the Euclidean distance of z-score data. Gene expression patterns are color-coded: red=up-regulation and green=down-regulation, along with age distributions and sickle cell trait status.

Table S5. Key resources table.

REAGENT or RESOURCE	SOURCE	IDENTIFIER
**Biological samples**		
Peripheral blood	Siaya County Referral Hospital	Not applicable
**Chemicals, peptides, and recombinant proteins**		
Giemsa Stain, Modified	Sigma-Aldrich	Cat # GS1L-1L
Trizol reagent	Thermo Fisher Scientific	Cat # 15596018
**Critical commercial assays**		
Unigold HIV kit	Trinity Biotech	Cat# 1206502
Determine HIV1/2 kit (100 tests)	Abbott Laboratories	Cat# 7D2347
**Deposited data**		
Raw Data, Metadata, Summary Data	National Library of Medicine (NLM) Gene Expression Omnibus (GEO)	GEO: GSE255403
Proteomics Data	Anyona *et al*[56]	N/A
**Oligonucleotides**		
Primer: GP40F1 Forward: TCTTAGGAGCAGCAGGAAGCACTATGGG	Yang *et al*[91]	N/A
Primer: GP41R1 Reverse: AACGACAAAGGTGAGTATCCCTGCCTAA	Yang *et al*[91]	N/A
Primer: GP46F2 Forward: ACAATTATTGTCTGGTATAGTGCAACAGCA	Yang *et al*[91]	N/A
Primer: GP47R2 Reverse: TTAAACCTATCAAGCCTCCTACTATCATTA	Yang *et al*[91]	N/A
**Software and algorithms**		
Spliced Transcripts Alignment to a Reference (STAR) version 2.5	Illumina® Inc.	N/A
HTSeq version 2.0	Anders *et al*[92]	N/A
SPSS® version 23.0	IBM SPSS Inc.	N/A
EdgeR version 3.16.5	Robinson *et al*[93]	N/A
Next-generation clustered heatmaps (NG-CHMs)	Ryan *et al*[94]	N/A
Metacore™	Clarivate Analytics	N/A
